# Functional characterization of an alkaline exonuclease and single strand annealing protein from the SXT genetic element of *Vibrio cholerae*

**DOI:** 10.1186/1471-2199-12-16

**Published:** 2011-04-18

**Authors:** Wen-yang Chen, John WS Ho, Jian-dong Huang, Rory M Watt

**Affiliations:** 1 Department of Biochemistry, The Chinese University of Hong Kong, Shatin, Hong Kong; 2 Department of Biochemistry, Li Ka Shing Faculty of Medicine, The University of Hong Kong, Pok Fu Lam, Hong Kong; 3 Oral Biosciences, Faculty of Dentistry, The University of Hong Kong, Prince Philip Dental Hospital, 34 Hospital Road, Hong Kong

## Abstract

**Background:**

SXT is an integrating conjugative element (ICE) originally isolated from *Vibrio cholerae*, the bacterial pathogen that causes cholera. It houses multiple antibiotic and heavy metal resistance genes on its ca. 100 kb circular double stranded DNA (dsDNA) genome, and functions as an effective vehicle for the horizontal transfer of resistance genes within susceptible bacterial populations. Here, we characterize the activities of an alkaline exonuclease (S066, SXT-Exo) and single strand annealing protein (S065, SXT-Bet) encoded on the SXT genetic element, which share significant sequence homology with Exo and Bet from bacteriophage lambda, respectively.

**Results:**

SXT-Exo has the ability to degrade both linear dsDNA and single stranded DNA (ssDNA) molecules, but has no detectable endonuclease or nicking activities. Adopting a stable trimeric arrangement in solution, the exonuclease activities of SXT-Exo are optimal at pH 8.2 and essentially require Mn^2+ ^or Mg^2+ ^ions. Similar to lambda-Exo, SXT-Exo hydrolyzes dsDNA with 5'- to 3'-polarity in a highly processive manner, and digests DNA substrates with 5'-phosphorylated termini significantly more effectively than those lacking 5'-phosphate groups. Notably, the dsDNA exonuclease activities of both SXT-Exo and lambda-Exo are stimulated by the addition of lambda-Bet, SXT-Bet or a single strand DNA binding protein encoded on the SXT genetic element (S064, SXT-Ssb). When co-expressed in *E. coli *cells, SXT-Bet and SXT-Exo mediate homologous recombination between a PCR-generated dsDNA fragment and the chromosome, analogous to RecET and lambda-Bet/Exo.

**Conclusions:**

The activities of the SXT-Exo protein are consistent with it having the ability to resect the ends of linearized dsDNA molecules, forming partially ssDNA substrates for the partnering SXT-Bet single strand annealing protein. As such, SXT-Exo and SXT-Bet may function together to repair or process SXT genetic elements within infected *V. cholerae *cells, through facilitating homologous DNA recombination events. The results presented here significantly extend our general understanding of the properties and activities of alkaline exonuclease and single strand annealing proteins of viral/bacteriophage origin, and will assist the rational development of bacterial recombineering systems.

## Background

The SXT mobile genetic element was originally isolated from an emerging epidemic strain of *Vibrio cholerae *(serogroup O139), which causes the severe diarrheal disease cholera [1]. Formerly referred to as a conjugative transposon, SXT is now classified as being a type of integrating conjugative element (ICE) [2,3]. Unlike bacteriophages and plasmids, ICEs cannot replicate their double stranded DNA (dsDNA) genomes autonomously. They integrate into the chromosome of the bacterial host, and replicate along with the host's chromosomal DNA. In response to certain physiological signals, they excise their genomic material and form a covalently closed circular double stranded (extrachromosomal) molecule [4]. SXT inserts its ca. 100 kb dsDNA genome into the 5'-end of the *prfC *gene on the *V. cholerae *chromosome in a site-specific manner [5]. After induction of the SOS response, SXT excises itself and re-circularizes into an extrachromosomal form which may be transferred by bacterial conjugation to recipient donor cells [3,6]. The genomic composition of SXT is closely related to that of R391, an ICE originally isolated from *Providencia rettgeri *(originally referred to as an IncJ element) [7], and they are fellow members of a large family of self-transmissible mobile genetic elements [3,4,8,9]. The SXT/R391 ICEs encode multiple proteins conveying resistance towards heavy metals (e.g. mercury) and antibiotics (e.g. sulfamethoxazole, trimethoprim, chloramphenicol and streptomycin) [2]. As such, they are efficient vehicles for the horizontal transfer of resistance genes within susceptible bacterial populations [3,6,8-11].

The SXT genome contains three consecutive coding DNA sequences (CDSs; *s064*, *s065 *and *s066*) arranged in an operon-like structure, which encode homologues of 'phage-like' proteins involved in DNA repair and/or recombination [2] (see Additional File 1 Panel A). The encoded S064 protein (SXT-Ssb) is highly homologous to bacterial single strand DNA (ssDNA) binding proteins (Ssb); S065 (SXT-Bet) is homologous to the Bet single stranded annealing protein (SSAP) from bacteriophage lambda (lambda-Bet, which is also referred to as a DNA synaptase or recombinase); and S066 (SXT-Exo) shares homology with the lambda Exo/YqaJ family of alkaline exonucleases [12,13] (See Additional File 1 Panel B). Related ICEs (e.g. R391, ICE*Vch*B33 and ICE*Pda*SpaI) all encode essentially identical *bet*, *exo *and *ssb *genes (>99% nucleotide identity) within highly similar genetic contexts [7,9,14].

Alkaline exonucleases are widely found in the genomes of viruses (especially herpesviridae), bacteriophages and other self-transmissible genetic elements [13,15]. The alkaline exonuclease from bacteriophage lambda (lambda-Exo) has been the subject of intense study since its discovery and isolation in the 1960s [16-25]. However, the *in vitro *activities of only one other closely-related homologue have been studied in any great detail; namely G34.1P from bacteriophage SPP1 (SPP1-Chu) [26,27]. The SPP1-Chu and lambda-Exo alkaline exonucleases both digest linear dsDNA molecules with strict 5'- to 3'- polarity. They bind to the termini of the dsDNA molecules and progressively hydrolyze the 5'-strand in a highly processive manner, releasing 5'-mononucleotides and generating long 3'-ssDNA tails [17,21,26,27] (see Additional File 2 for a schematic overview). The partnering SSAP protein (lambda-Bet or G35P, respectively) coats the nascent 3'-ssDNA tails, forming helical nucleoprotein filaments [28,29], and promotes their annealing with complementary regions of (partially) single stranded DNA on the bacteriophage, episome or host chromosome [30-32]. This 'strand annealing' pathway may occur at double strand breaks (DSBs) or at the replication fork. Other host cell DNA recombinases (synaptases) such as RecA may also play a role in recombination events, and strand invasion of an intact DNA duplex may, or may not occur [20,32-34]. DNA repair and replication proteins from the bacterial host cell then process and resolve the branched DNA intermediates formed [20,33]. The alkaline exonuclease may also 'trim' any ssDNA overhangs formed after SSAP-mediated annealing events, enabling the resultant nicks to be closed by DNA ligase [21,23].

Aside from their native biological roles, the activities of partnering SSAP and alkaline exonuclease proteins (e.g. lambda-Bet/Exo, RecET and Che9c gp60/61) have attracted increasing interest for their use in bacterial *in vivo *DNA homologous recombination-based genetic engineering procedures [35-41]. In an approach referred to as 'recombineering', partnering SSAP and alkaline exonuclease proteins co-expressed within a bacterial host cell efficiently mediate homologous DNA recombination (genetic crossover) between PCR-generated linear dsDNA molecules and the desired chromosomal or episomal target, via short regions (ca. 35-50 bp) of shared sequence homology. Phenotypic changes or selectable markers e.g. antibiotic resistance genes are generally required to detect recombination events. Although the precise mechanism has not yet been established, it appears that only partnering alkaline exonuclease and SSAP proteins (i.e. ones from the same biological system) can associate with each other and function together to promote DNA recombination; e.g. lambda-Exo does not bind to, or functionally-cooperate with the *E. coli *RecT protein [42].

Genetic studies have recently revealed that the SXT-Bet (S065) and SXT-Exo (S066) proteins promote DNA recombination between two different ICE molecules residing within the same heterologous host cell (*Escherichia coli*), leading to the formation of 'hybrid' ICEs [10]. Furthermore, Datta *et al*. [39] have recently shown that SXT-Bet can efficiently promote genetic recombination between single stranded oligonucleotides and the *E. coli *chromosome. This prompted us to characterize the biochemical and biological activities of the SXT-Exo and Bet proteins, to help establish their proposed involvement in DNA recombination events.

Here, we show that SXT-Exo is a processive alkaline exonuclease with the ability to digest linear, but not circular ssDNA and dsDNA molecules. We further demonstrate that SSAP and Ssb proteins stimulate the *in vitro *dsDNA exonuclease activities of SXT-Exo. When expressed from plasmids established in *E. coli *cells, the SXT-Exo and SXT-Bet proteins mediate the homologous recombination of PCR-generated dsDNA fragments with the chromosome via short flanking regions of shared sequence homology.

## Methods

### Materials

All oligonucleotides were purchased from TechDragon Ltd. (Shatin, Hong Kong) in a desalted (Bio-RP) form. Polymerase chain reaction (PCR) was performed using Expand DNA polymerase and buffers (Roche), containing PCR-grade deoxynucleotide triphosphates (dNTPs, Roche), on a GeneAmp 9700 thermal cycler (Applied Biosystems). Plasmids were purchased from Novagen and Invitrogen. Restriction enzymes were purchased from New England Biolabs (NEB), and digestions were performed according to the manufacturer's instructions. DNA was routinely analyzed after electrophoresis on 1% agarose (Invitrogen) in Tris-acetate EDTA (TAE, USB) gels. Visualization and quantification of DNA bands (densitometry) was performed after staining with ethidium bromide (Sigma) using UV trans-illumination on a ChemiDoc XRS molecular imaging system with Quantity One v4.6.6 software (BioRad). Plasmid DNA was routinely purified from 4 ml (stationary phase) overnight cultures using Qiaprep spin miniprep kits (Qiagen). PCR products and DNA excised from agarose gels were purified using Qiaquick PCR purification and gel extraction kits (Qiagen), respectively. Where applicable, linearized DNA was dephosphorylated using calf intestinal phosphatase (NEB), according to the manufacturer's instructions, then gel-purified. DNA and protein alignments, sequence analysis, primer design, etc. were performed using Omiga v2.0 (Oxford Molecular). Graphs were prepared using OriginPro 7.5 SR1 (OriginLabs).

### Strains, plasmids and Media

Plasmid pJB1 was constructed by Dr. John Beaber and was generously supplied by Prof. Matthew Waldor (Harvard Medical School and HHMI). pJB1 contains the region of the SXT integrating conjugative element (ICE) that includes the *ssb (s064)*, *bet (s065) *and *exo (s066) *genes, was used as the template for the PCR-amplification of all SXT genes. Plasmid pBAD-ETγ [35] was generously supplied by Prof. A. Francis Stewart (TU Dresden). All arabinose-inducible plasmids constructed here are derivatives of pBAD-ETγ (see Additional File 3), and contain identical 4503 bp NcoI/HindIII backbone fragments, which houses the ColE1 *ori*, *bla *ampicillin resistance gene, *araC *repressor, P_BAD _operator/promoter region, and ribosome binding site immediately upstream of the adjacent NcoI and NdeI restriction sites.

All gene targeting, gene cloning and plasmid propagation procedures were performed in *E. coli *DH10B [Invitrogen, genotype: F- *mcr*A Δ(*mrr*-*hsd*RMS-*mcr*BC) Φ80*lac*ZΔM15 Δ*lac*X74 *rec*A1 *end*A1 *ara*D139 Δ(*ara leu*) 7697 *gal*U *gal*K *rps*L *nup*G λ-], incubating plates and liquid cultures at 37°C. All oligonucleotides, linear dsDNA and plasmid DNA were transformed into *E. coli *cells by electroporation using a MicroPulser electroporator with 1 mm gap electroporation cuvettes (BioRad). Transformed cells were plated onto Luria-Bertani (LB) agar (USB), and liquid cultures were grown in LB medium (USB); supplementing with kanamycin (Kan, 50 μg/ml, USB), ampicillin (Amp, 50 μg/ml, USB) and/or chloramphenicol (Cm, 30 μg/ml, Sigma) for plasmid maintenance, where appropriate. LB-agar containing chloramphenicol (12 μg/ml) was used to select for *E. coli *strains containing chromosome-based copies of the chloramphenicol (*cat*) resistance gene. Protein expression was performed in *E. coli *BL21 (DE3) or BL21 (DE3) pLysS Rosetta (Novagen); inducing expression by the addition of isopropyl-1-thio-β-D-galactopyranoside (IPTG, USB) to between 0.1 to 0.5 mM; incubating cultures post-induction at 20-37°C.

### Plasmid construction

The *SXT-exo *(s066) gene [GenBank:AY055428.1, 73921 - 74937] was PCR amplified from plasmid pJB1, using the Sexofor (TATACATATGAAGGTTATCGACCTATCAC) and SexoRevX (TTAACTCGAGTTAAAAATAAAATGAGCTCGGCGA) primers. After NdeI/XhoI digestion (restriction sites underlined), it was cloned into pET28a (Novagen) to create pEA1-1. The *lambda-exo *gene was PCR-amplified from bacteriophage lambda *cI857 ind*1 *Sam7 *DNA (NEB) using the exofor1 (TATAACATATGACACCGGACATTATCCTG) and exorev3 (TTATCTCGAGTCGCCATTGCTCCCCAAA) primers, and cloned via NdeI/XhoI into pET32a (Novagen) to create pEE4. The *SXT-bet *(s065) gene [GenBank:AY055428.1, 72817 - 73635] was PCR amplified from plasmid pJB1 using the SXTNdeI (TTAACATATGGAAAAACCAAAGCTAATCCAA) and SXTXhoI (TATACTCGAGCTAAGAAGCTAAAGGCTGTGTGAG) primers, then cloned via NdeI/XhoI into pET28a to create plasmid pX28-1 (N-terminal His-T7 tagged SXT-Bet). The *SXT-ssb *(*s064*) gene [GenBank: AY055428.1, 72318 - 72737] was PCR amplified from plasmid pJB1 using the SSBfor1 (TATAGAATTCACC**ATG**GGAAACCAAGTAACACTCATAGGC) and SSBrevX (TTAACTCGAGTTA AAAATCTGGTTCAGGATAAGTTTG) primers, then cloned via EcoRI/XhoI into pET28a to create plasmid pSB2. The *lambda-bet *gene was PCR-amplified from bacteriophage lambda *cI857 ind*1 *Sam7 *DNA (NEB) using the betfor1 (TATAACATATGAGTACTGCACTCGCAACG) and betrev2 (TATTCTCGAGTGCTGCCACCTTCTGCTCTG) primers, digested with NdeI/XhoI and cloned into pET32a to create p1DB.

Plasmids pB1E4, pBex4b1, pBX2B and pBAD-28MCS are all derivatives of plasmid pG5P4-1, which contains the 4503 bp NcoI/HindIII 'backbone' of the pBAD-ETγ plasmid [35]. First, pG1.2A was created by PCR-amplifying the cloning the *gam *gene from bacteriophage lambda *cI857 ind*1 *Sam7 *using the gamfor1 (TATAACATATGGATATTAATACTGAAACT) and gamrev2 (ATATTCTCGAGTACCTCTGAATCAATATC) primers, then cloning it into pET32a via NdeI/XhoI. Then, the *gam *gene and downstream hexahistidine tag were PCR amplified from pG1.2A using the gamLbad1 (TATGCC*ATGGGCCATATG*GATATTAATACTGAAACTGAG) and XHh6 (TACCAAGCTTAGTGGTGGTGGTGGTGGTG) primers, the ca. 425 bp PCR product was digested with NcoI/HindIII, and then ligated with the 4503 bp NcoI/HindIII backbone of pBADETγ to create pG5P4-1. All genes were cloned into pG5P4-1 in such a way as to replace the *gam *gene. All reverse PCR primers included a stop codon preceding the XhoI site, so that they did not encode C-terminal hexahistidine fusions).

The contiguous *bet *and *exo *genes from bacteriophage lambda *cI857 ind*1 *Sam7 *were PCR amplified using the betfor1 and exorev4 (TTATCTCGAGTCATCGCCATTGCTCCCCAA) primers. The ca. 1470 bp PCR product was digested with Nde/XhoI and cloned into plasmid pG5P4-1 to create plasmid pB1E4 (placing them both under the control of a P_BAD _promoter immediately upstream of *bet*). The region of SXT containing adjacent *SXT-bet *and *SXT-exo *genes (GenBank: AY055428.1, 72817 - 74937) was PCR amplified from pJB1 using the SXTNdeI and SexorevX primers; then the ca. 1590 bp PCR product was digested with NdeI/XhoI and cloned into pG5P4-1 to create plasmid pBex4b1 (placing the SXT *bet*-*exo *genes under the control of a P_BAD _promoter immediately upstream of SXT-*bet*).

The region of SXT containing adjacent *SXT-ssb*, *bet *and *exo *genes (GenBank: AY055428.1, 72318 - 74937) was PCR amplified from pJB1 using the Ssbfor1 and SexorevX primers; then the ca. 2630 bp PCR product was digested with EcoRI/XhoI and cloned into pG5P4-1 to create plasmid pBX2B (placing the SXT *ssb*-*bet*-*exo *genes under the control of a P_BAD _promoter immediately upstream of SXT-*ssb*). For a control plasmid, the ca. 140 bp multiple cloning site from pET28a was excised by digestion with NcoI/XhoI, and was cloned into pG5P4-1 to create plasmid pBAD-28MCS.

### Protein expression, purification and analysis of multimericity

*E. coli *BL21 (DE3) pLys Rosetta containing pEA1-1 was grown in LB medium containing 50 μg/ml kanamycin at 37°C to an OD_600 _of ~0.60; IPTG (0.4 mM) was added, then cultures were incubated at 30°C for 6 hours. The washed cell pellet was resuspended in lysis buffer (25 mM Tris-HCl pH7.4, 500 mM NaCl, 25 mM imidazole) then lysed by sonication. After centrifugation (15,000 g, 30 min; 0.45 μm filtered) the supernatant was applied to a 5 ml Hitrap Chelating FF column (GE Healthcare) that had been charged with nickel (II) ions. His-tagged SXT-Exo was eluted using a linear gradient of imidazole in lysis buffer (25-500 mM). Fractions containing pure protein were pooled, and buffer was exchanged using a G-25 Sephadex desalting column (GE Healthcare) pre-equilibrated with 25 mM Tris-HCl pH7.4, 50 mM NaCl. The SXT-Bet, SXT-Ssb, lambda-Exo and lambda-Bet proteins were analogously expressed from their respective pET-based expression vectors in BL21 (DE3), grown in LB medium containing 50 μg/ml kanamycin or 100 μg/ml ampicillin (as appropriate), and were similarly purified by immobilized Ni-ion affinity chromatography, followed by buffer exchange on Sephadex G-25 (GE Healthcare).

The multimeric arrangements of the purified, recombinant SXT-Exo and lambda-Exo proteins (100 μl) were analyzed using a Tricorn Superdex 200 HR 10/300 GL column on an AKTA-FPLC (GE Healthcare) pre-equilibrated with gel filtration buffer (25 mM Tris-HCl pH7.4, 150 mM NaCl, 1 mM EDTA, 5 mM imidazole); at a flow-rate of 0.4 ml/min at 4°C, monitoring the eluent at 280 nm. The column was pre-calibrated with the following protein standards (GE healthcare): ferritin (440 kDa); aldolase (158 kDa); thyroglobulin (67 kDa); ovalbumin (43 kDa); chymotrypsinogen A (25 kDa) and ribonuclease A (13.7 kDa).

### Qualitative determination of SXT-Exo substrate range, mode of digestion and metal ion dependence

#### DNA substrate determination

Assay mixtures (20 μl) containing SXT-Exo (0.6 μg, 5 pmol of trimers) and a DNA substrate: i) undigested pUC18 (184 ng); ii) PstI-linearized pUC18 (184 ng); iii) dephosphorylated PstI-linearized pUC18 (184 ng); or iv) M13-phage ssDNA (TaKaRa, 300 ng, 0.13 pmole) in Tris-HCl (25 mM, pH7.4), 50 mM NaCl with/without 10 mM MgCl_2 _(as indicated in the text), were incubated at 37°C for 30 minutes then quenched (20 mM EDTA). Aliquots (20 μl) were analysed on 1% TAE agarose gels.

#### Determination of digestion polarity

A representative linear double stranded DNA substrate ('unmodified'; 712 bp) was synthesized by PCR using pET32a as a template, with the primers T7for (TAATACGACTCACTATAGGG) and T7rev (GCTAGTTATTGCTCAGCGG). An analogous 712 bp dsDNA substrate ('PT-modified') was similarly synthesized by PCR, using the phosphorothioate modified primers T7forPT3 (TAATACGACTCACTATA_S_G_S_G_S_G) and T7revPT3 (GCTAGTTATTGCTCAG_S_C_S_G_S_G), where the subscript 'S' denotes a (nuclease resistant) phosphorothioate linage instead of a normal phosphodiester lingage. Both PCR products were 5'-phosphorylated using T4 polynucleotide kinase (NEB), then purified (QIAquick PCR purification kit) prior to use. The 5'-phosphorylated 'unmodified' and 'PT-modified' dsDNA substrates (0.1 mg) were separately incubated at 37°C with lambda-Exo (3 μg) or SXT-Exo (30 μg) in Tris-HCl, (25 mM, pH7.4), 50 mM NaCl, 10 mM MgCl_2 _(total volume 40 μl). Aliquots (20 μl) were quenched (20 mM EDTA + 1% SDS) immediately and after 30 mins, and analyzed on 1% agarose TAE gels.

#### Time course analysis

Assay mixtures (250 μl) containing NdeI-linearized pET28a (1.84 μg, 0.56 pmol) and SXT-Exo (6 μg, 50 pmol of trimers) in Tris-HCl (25 mM, pH7.4), 50 mM NaCl, 10 mM MgCl_2 _were incubated at 37°C. Aliquots (20 μl) were quenched (1% SDS + 20 mM EDTA) at the times indicated over a 160 min period, then analyzed on 1% agarose TAE gels.

### Quantitative determination of double strand DNA exonuclease activities by quenched PicoGreen fluorescent assays

The double strand exonuclease activities of SXT-Exo and lambda-Exo under various conditions were determined by quantifying the amounts of double strand DNA that remained after enzymatic incubation, using the PicoGreen DNA fluorescence reagent (Invitrogen, catalogue # P7589). These quenched PicoGreen fluorescence assays were performed as described previously [26,43] with minor modifications. All experiments described below utilized analogous assay procedures, which were performed in 96-well microtitre plates (Iwaki 3860-096, Asahi Glass Co., Japan). Four to six independent replicates were performed for each experimental condition; with graphs showing the mean values ± standard deviation. Unless otherwise stated, assays (48 μl) contained SXT-Exo protein (2 pmol of trimers) in exonuclease buffer (25 mM Tris-HCl pH7.4, 50 mM NaCl, 0.5 mM MnCl_2_). Assays were initiated by the addition of 2 μl of a solution of the DNA substrate, typically PstI-linearized pUC18 (5 ng, 0.003 pmol), in exonuclease buffer; and were incubated at 37°C for 30 mins, before quenching by the addition of EDTA to a final concentration of 20 mM. PicoGreen reagent was then added (250-fold dilution) and fluorescence levels were immediately measured (excitation 485 nm/emission 535 nm) using a Perkin Elmer 1420 multi-label counter. The degree of dsDNA digestion was determined using: (N-R)/(N-P); where N = fluorescence reading for the starting DNA concentration; R = fluorescence reading at the quenched time point; P = fluorescence reading obtained for half of the concentration of linear dsDNA substrate used in the assay, which had been heat-denatured (P is an approximation of the digestion end-point; for most assays, P = fluorescent reading for 2.5 ng of heat-denatured PstI-linearized pUC18 in 50 μl of exonuclease buffer).

### Determination of optimal conditions for SXT-Exo double strand DNA exonuclease activities

Quenched PicoGreen fluorescence assays were performed, quenched and analyzed as described above with minor modifications. Determination of optimal Mg^2+ ^and Mn^2+ ^ion concentrations: Assays (50 μl) contained SXT-Exo (2 pmol of trimers), PstI-linearized pUC18 (5 ng, 0.003 pmol) in Tris-HCl (25 mM, pH7.4), 50 mM NaCl containing MnCl_2 _(0-10 mM) or MgCl_2 _(0-50 mM) at the concentrations indicated in the text; and were incubated at 37°C for 30 mins. Determination of optimal pH: Assays (50 μl) contained SXT-Exo (2 pmol of trimers), PstI-linearized pUC18 (5 ng, 0.003 pmol), 50 mM Tris-HCl, 50 mM NaCl, 0.5 mM MnCl_2_; adjusted to the appropriate pH value (pH7.0 - pH9.0); and were incubated at 37°C for 30 mins. Determination of optimal temperature: Assays (50 μl) contained SXT-Exo (6 pmol of trimers), PstI-linearized pUC18 (5 ng, 0.003 pmol) in exonuclease buffer. All solutions were pre-equilibrated at the temperature indicated in the text (31-47°C) using a thermostat-regulated water bath; an incubation time of 1 minute was used. Effects of adding various concentrations of monovalent or divalent metal cations: Assays (50 μl) contained SXT-Exo (2 pmol of trimers), PstI-linearized pUC18 (5 ng, 0.003 pmol) in Tris-HCl (25 mM, pH7.4), 50 mM NaCl, 0.5 mM MnCl_2_; as well as the salt indicated in the text (NaCl, KCl, CaCl_2_, Na_2_SO_4_, K_2_SO_4 _or Na_2_HPO_4_; to a final concentration between 0-500 mM); and were incubated at 37°C for 30 mins. The relative dsDNA exonuclease activities were calculated (as a percentage) by comparison with results from analogous assays that contained: SXT-Exo (2 pmol of trimers), PstI-linearized pUC18 (5 ng, 0.003 pmol) in Tris-HCl (25 mM, pH7.4), 50 mM NaCl, 0.5 mM MnCl_2_.

### Double strand DNA end preference

Assay mixtures contained SXT-Exo (12 pmol of trimers) and 30 ng of the linear double strand DNA substrate indicated in the text: PstI-linearized pUC18, BamHI-linearized pUC18, SspI-linearized pUC18, or fully 5'-dephosphorylated PstI-linearized pUC18; in exonuclease buffer (300 μl). Assays were incubated at 37°C for 30 mins. 50 μl aliquots were removed and quenched (20 mM EDTA) after 1, 2, 5, 10, 20 and 40 minutes; and levels of dsDNA were immediately quantified using PicoGreen reagent as described above. Data were fitted to hyperbolae using Origin v7.5 SR1. Figures show the mean values obtained (6 replicates) and omit the standard deviation for reasons of clarity.

### Determination of double strand DNA end preference for SXT-Exo and lambda-Exo using annealed oligonucleotide substrates

The 50Cy3 (CAgTCACgACgTTgTAAAACgACggCCAgTgCCAAgCTTgCATgCCTgCA-Cy3) and 70Cy3 (TTTTTTTTTTTTTTTTTTTTCAgTCACgACgTTgTAAAACgACggCCAgTgCCAAgCTTgCATgCCTgCA-Cy3) oligonucleotides both contain Cy3 fluorescent groups at their 3'-termini, and were phosphorylated at their 5'-termini using T4 polynucleotide kinase (Invitrogen) according to the manufacturer's instructions, then purified (Qiaquick PCR purification kit, Qiagen). The 50blunt (TgCAggCATgCAAgCTTggCACTggCCgTCgTTTTACAACgTCgTgACTg) and 70overhang (TgCAggCATgCAAgCTTggCACTggCCgTCgTTTTACAACgTCgTgACTgTTTTTTTTTTTTTTTTTTTT) oligonucleotides do not contain 3'-Cy3 groups, and were not 5'-phosphorylated. Three partially double stranded DNA substrates were prepared by thermally annealing various oligonucleotide pairs: "Blunt ended" = 5'-PO_4_-50Cy3 + 50blunt; "5'-overhang" = 5'-PO_4_-70Cy3 + 50blunt; and "3'-overhang" = 5'-PO_4_-50Cy3 + 70overhang (see Additional File 4 Panel B). With the careful exclusion of light, equimolar amounts of each oligonucleotide in millipure water were heated to 100°C for 5 minutes, then allowed to cool slowly to room temperature. Substrates were used without further purification. Exonuclease assays: Reaction mixtures (50 μl) contained: i) SXT-Exo (50 pmol of trimers), Tris-HCl (50 mM, pH 8.0), 0.5 mM MnCl_2_, and 20 pmol of one of the Blunt ended, 5'-overhang or 3-overhang annealed oligonucleotide substrates; or ii) lambda-Exo (3 pmol of trimers) in Tris-HCl (50 mM, pH 8.0), 5 mM MgCl_2_, and 20 pmol of one of the Blunt ended, 5'-overhang or 3'-overhang annealed oligonucleotide substrates. Assays were incubated at 25°C, and 10 μl aliquots were withdrawn and quenched with 40 μl of gel loading buffer (50 mM Tris pH8.0, 5 mM EDTA, 1% SDS, 8 M urea, 20% glycerol) at the times indicated in the text (0-20 minutes for SXT-Exo; 0-10 minutes for lambda-Exo). Samples were stored on ice in the dark prior to resolution on denaturing polyacrylamide gels [7 M urea-TBE polyacrylamide (12%, 37.5:1)]. Gels were scanned for fluorescence using a Typhoon™ 9410 Variable Mode Imager (excitation 532 nm/emission 580 nm). Band intensities on the scanned gel images were quantified using Quantity One software (BioRad) to calculate the degree of digestion of the (annealed) 5'-PO_4_-70Cy3 or 5'-PO_4_-50Cy3 oligonucleotides. Data were fitted to hyperbolae using Origin v7.5 SR1. Figures show the mean values obtained (4 replicates) and omit the standard deviation for reasons of clarity. In separate experiments, 8 pmol; 4 pmol; 2 pmol; 1 pmol; 0.5 pmol; 0.25 pmol; 0.125 pmol and 0.0625 pmol of the 5'-PO_4_-70Cy3 and 5'-PO_4_-50Cy3 oligonucleotides were resolved on 7 M urea-TBE polyacrylamide gels, and analogously scanned for fluorescence to construct standard curves (see Additional File 4 Panels C and D).

### Determination of exonuclease processivity and rate

The procedure used was analogous to that previously described by Myers and co-workers [24,26]. SXT-Exo (41 nmol of trimers) and PstI-linearized pUC18 DNA (30 ng, 18.1 pmol) in 150 μl of Tris-HCl (25 mM, pH7.4), 50 mM NaCl were pre-incubated for 5 mins at 25°C. Reactions were initiated by the addition of 50 μl of Tris-HCl (25 mM, pH7.4), 50 mM NaCl, 2 mM MnCl_2 _(making the final concentration of Mn^2+ ^ions 0.5 mM) and were incubated at 25°C. After 30 s, unbound SXT-Exo was 'trapped' by the addition of heparin (150 μg; Sigma, Cat. #H3393) in exonuclease buffer (100 μl). 50 μl aliquots were withdrawn at various time points (0-30 mins) and quenched with EDTA (20 mM). Levels of dsDNA were immediately quantified using PicoGreen reagent as described above. Analogous control experiments using a 'trapping' buffer without heparin (i.e. 100 μl of exonuclease buffer) were performed. The number of nucleotides digested was determined by calculating the percentage of DNA substrate digested at each time point, then multiplying this value by 1343; which is the number of nucleotides available for digestion per DNA strand on the linearized (2686 bp) pUC18 molecule, assuming digestion occurs from both termini. Four independent replicates of each experiment were conducted, and graphs show the mean values ± standard deviation. To obtain the processivity and initial rate of DNA digestion at 25°C, data from the heparin trap experiments was fitted to a hyperbola using Origin v7.5 SR1 as previously described [24].

### Protein-mediated modulation of the double stranded DNA exonuclease activities of SXT-Exo and lambda-Exo

SXT-Exo (2 pmol of trimers), and 2 pmol of the protein indicated in the text [BSA (Sigma), SXT-Bet, SXT-Ssb or lambda-Bet] in 48 μl of exonuclease buffer were incubated at 25°C for 5 mins. PstI-linearized pUC18 (5 ng, 0.003 pmol) in 2 μl of exonuclease buffer was added to initiate the reaction, incubating assays at 25°C for 30 mins before quenching (20 mM EDTA). Levels of dsDNA were immediately quantified using PicoGreen reagent as described above. In analogous experiments, lambda-Exo (2 pmol of trimers) and 2 pmol of the protein indicated in the text [BSA, SXT-Bet, SXT-Ssb or lambda-Bet], in 48 μl of Tris-HCl (25 mM, pH7.4), 50 mM NaCl, 5 mM MgCl_2 _were incubated at 25°C for 5 mins. PstI-linearized pUC18 (5 ng, 0.003 pmol) in 2 μl of Tris-HCl (25 mM, pH7.4), 50 mM NaCl, 5 mM MgCl_2 _was added to initiate the reaction, incubating assays at 25°C for 10 mins before quenching and analyzing dsDNA levels using PicoGreen reagent. Six replicates of each experiment were performed, and data was analyzed using ANOVA (SPSS software package). Figures show the mean values ± standard deviation, with corresponding P values indicated (compared to control reaction where no protein was added).

### Oligonucleotide exonuclease assays

A 75-mer of oligothymidine (dT_75_) was used directly, or where applicable, was phosphorylated at the 5'-terminus (5'-P-dT_75_) using T4 polynucleotide kinase (Invitrogen) according to the manufacturer's instructions. Assays (250 μl) containing dT_75 _or 5'-P-dT_75 _(400 pmol) in Tris-HCl (25 mM, pH7.4), 50 mM NaCl, 10 mM MgCl_2 _were initiated by the addition of SXT-Exo (100 pmol of trimers). Reactions were quenched immediately (20 mM EDTA) or after incubation at 37°C for 3 or 25 minutes. Protein was removed after precipitation by consecutive heating (100°C, 10 mins), cooling (4°C, 10 mins), and centrifugation (15.6 kg, 5 mins). Supernatant (100 μl) was analyzed by size exclusion chromatography using a Tricorn Superdex 200 HR 10/300 GL column pre-equilibrated with 25 mM Tris-HCl pH7.4, 150 mM NaCl, 1 mM EDTA; at a flow-rate of 0.4 ml/min at 4°C, monitoring the elution of oligonucleotides at 254 nm. A standard curve for dT_75 _was constructed (loading 10, 20, 40, 60 or 80 nmoles of oligonucleotide onto the gel filtration column under analogous conditions; see Additional File 4 Panel A). The area under the main peak on the gel filtration chromatogram (elution volume 12.9 ml), was proportional to the amount of dT_75 _present.

### Determination of double strand DNA recombination activities

A chloramphenicol resistance (*Cm^r^*) cassette was PCR-amplified from plasmid pEGFP-loxP-CmR-loxP [44] using primers ECgalKF1 (GTTTGCGCGCAGTCAGCGATATCCATTTTCGCGAATCCGGAGTGTAAGAATAAAAATAGGCGTATCACGAG) and ECgalKR1 (TTCATATTGTTCAGCGACAGCTTGCTGTACGGCAGGCACCAGCTCTTCCGTAGTGAACCTCTTCGAGGGAC), to create a dsDNA molecule (*Cm<>galK*) that contained 50 bp flanking regions with sequence homologous to the 5'- and 3'-ends of the *E. coli galK *gene (underlined in the two primer sequences; see Figure 11 Panel A). These primers were analogous to those previously described by Yu *et al*. [38], which were used to perform analogous *galK *gene targeting experiments with the lambda-Red proteins. The *Cm<>galK *PCR product was gel-purified (Qiaquick Gel Extraction Kit, Qiagen); DNA was eluted from the spin column with 1 mM Tris-HCl (pH8.0). Plasmids pBAD-ETγ, pB1E4A, pBex4b1, pBX2B and pBAD-28MCS were separately transformed into *E. coli *DH10B by electroporation. Individual transformant colonies (LB-agar + ampicillin 50 μg/ml) were inoculated into LB medium containing 50 μg/ml ampicillin (5 ml), which were incubated with shaking at 37°C overnight. Aliquots from the overnight cultures were expanded 1:100 in fresh LB medium containing 50 μg/ml ampicillin (50 ml in 250 ml conical flasks), and were incubated with shaking (37°C, 60 mins). Expression was induced by the addition of filter-sterilized aqueous arabinose solution (10% w/v) to a final concentration of 0.1%, and flasks were incubated with shaking (37°C, 60 mins). Flasks were then rapidly chilled in ice-water (with swirling) for 20 minutes, cultures were decanted into pre-chilled 50 ml Falcon tubes, and cells were pelleted by centrifugation (4000 g, 10 mins, 1-3°C). After carefully decanting the medium, cell pellets were gently washed with ice-cold sterile water (40 ml), and centrifuged as before. Washed cell pellets were resuspended in ice-cold sterile water (190 μl) and transferred to pre-chilled 1.5 ml microcentrifuge tubes placed in wet-ice. The purified *Cm<>galK *dsDNA targeting cassette (1.18 μg) was added to each tube, and after 2-3 minutes, aliquots of the resuspended cell pellets (2 × 90 μl) were pipetted into (pre-chilled) electroporation cuvettes placed on ice. Immediately after electroporation (1.8 kV), LB medium (1 ml, no antibiotics) was added to the electroporation cuvettes, and the resuspended cell mixtures were transferred to 1.5 ml microcentrifuge tubes, and were allowed to recover (37°C, 90 mins). A 10 μl aliquot was removed, diluted 1:10,000 in LB media then plated onto LB-agar in order to calculate the surviving cell count. The remaining ca. 1 ml of transformed cell suspension was centrifuged (12,000 g, 1 min) then the entire cell pellet was plated onto LB-agar containing 12 μg/ml chloramphenicol. Agar plates were incubated at 37°C for 16 hours prior to colony counting. The recombination efficiency was calculated by dividing the number of chloramphenicol-resistant colonies by the number cells surviving electroporation (colony forming units). 10 Cm-resistant colonies from each plate were screened by PCR, to check that the *Cm^r ^*cassette had been chromosomally-integrated in the correct manner. All experiments were performed in duplicate, and repeated 4 times (8 replicates). Errors were calculated on the mean values ± standard deviation.

## Results

### SXT-Exo forms a trimer analogous to lambda-Exo

**Figure 1:**
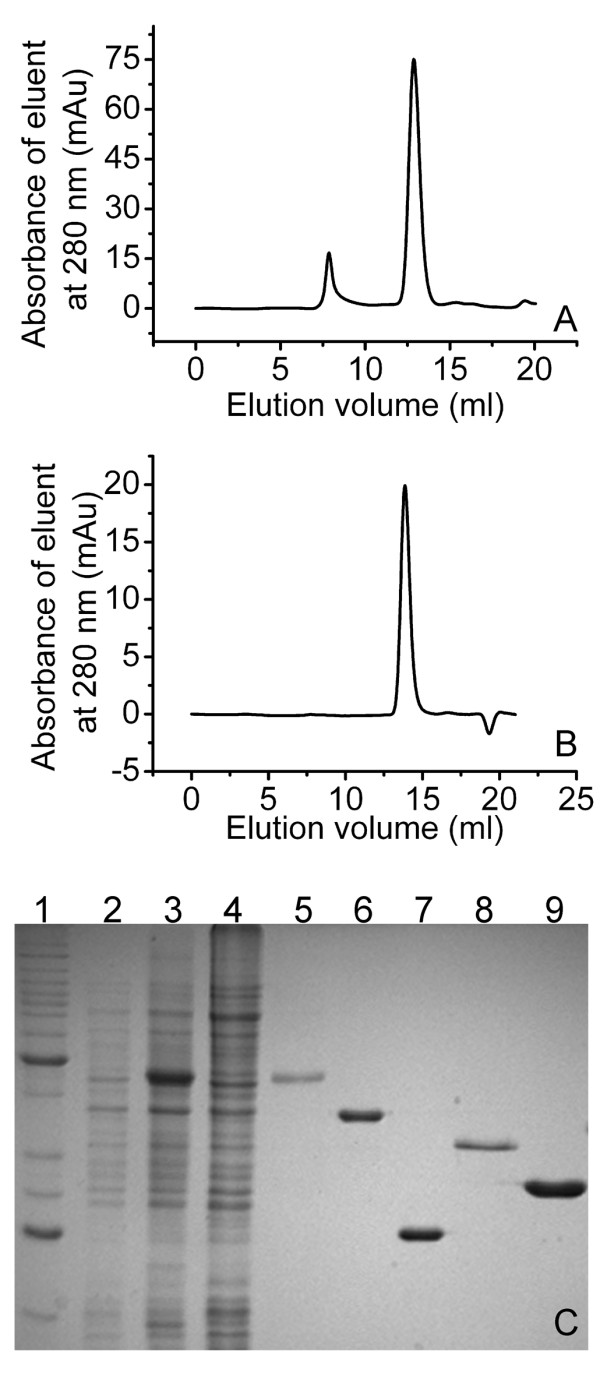
**Purification of SXT-Exo and lambda-Exo, and determination of their multimericity by size exclusion chromatography**. Panel** A**: Size exclusion chromatogram of purified SXT-Exo protein expressed from plasmid pEA1-1. Panel** B**: Size exclusion chromatogram of purified lambda-Exo protein expressed from plasmid pEE4. Panel** C**: 12% polyacrylamide gel (SDS-PAGE) analysis of the SXT-Exo purification procedure and purified SXT-Bet, SXT-Ssb, lambda-Bet and lambda-Exo proteins; lane 1: Benchmark protein ladder (Invitrogen); lane 2: pEA1-1/*E. coli *BL21 (DE3) pLysS Rosetta whole cell extract immediately prior to induction; lane 3: whole cell extract 6 hours after induction with IPTG; lane 4: supernatant from cell extract 6 hours post induction; lane 5: purified SXT-Exo; lane 6: purified SXT-Bet expressed from pX28-1; lane 7: purified SXT-Ssb expressed from pSB2; lane 8: purified lambda-Bet expressed from p1DB; lane 9: purified lambda-Exo expressed from pEE4.

The *SXT-exo *(*s066*), *SXT-bet (s065) *and *SXT-ssb (s064) *genes were cloned into pET28a vectors (Novagen) and expressed in *E. coli *as 20 amino acid N-terminal T7-hexahistidine fusions (*MGSSHHHHHHSSGLVPRGSH***M**...). The *bet *and *exo *genes from bacteriophage lambda were cloned into pET32a vectors (Novagen) and expressed in *E. coli *as 8aa C-terminal hexahistidine fusions (...LEHHHHHH*). All five of the recombinant His-tagged proteins were purified by immobilized Ni-affinity chromatography followed by desalting, and were determined to be 95-99% pure by SDS-PAGE (Figure 1 panel C). With the exception of SXT-Exo, the purified recombinant proteins all exhibited good long-term physical stability in a variety of buffer systems (data not shown). Consequently, all experiments involving SXT-Exo were performed using batches of freshly-expressed and purified protein.

The multimericity of the recombinant SXT-Exo and lambda-Exo proteins were determined using size exclusion chromatography (Figure 1). Two peaks were apparent in the SXT-Exo chromatogram (Panel A). SDS-PAGE analysis of eluted fractions corresponding to both peaks confirmed that they contained only SXT-Exo protein (data not shown). The minor peak (eluting at 7.9 ml) corresponded to a molecular weight of ca. 1350 kDa, which equated to ca. 33 SXT-Exo monomers. The major peak (eluting at 12.9 ml) correlated to a molecular weight of 145 kDa, which corresponded to ca. 3.5 SXT-Exo monomers. Lambda-Exo eluted at 13.8 ml (Panel B), correlating to a molecular weight of 91 kDa, which corresponded to 3.35 protein monomers. This indicated that the recombinant lambda-Exo and SXT-Exo proteins both formed stable trimers in solution, with no monomeric forms detected. This strongly suggests that SXT-Exo adopts a 'doughnut-shaped' homotrimeric arrangement analogous to that of lambda-Exo [19]. The torroidal trimers of both these two proteins possibly migrate faster through the gel-filtration column due to their flattened, non-spherical topology (i.e. larger Stokes radius). The high molecular weight, soluble aggregation of ca. 33 SXT-Exo protein monomers is most likely an artefact due to heterologous over-expression in *E. coli*, and is probably not biologically relevant. Consequently, in all subsequent biochemical assays, the concentrations of the SXT-Exo and lambda-Exo proteins were reported in terms of moles of trimers, as this is most likely the active form of both exonucleases.

### Qualitative determination of SXT-Exo nuclease activities

**Figure 2:**
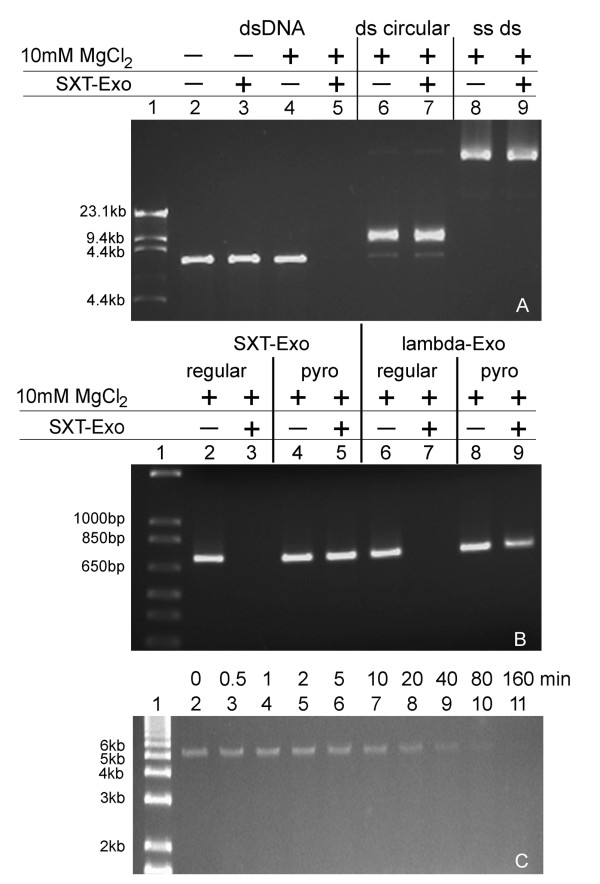
**Qualitative analysis of the metal ion dependence, DNA substrate preferences and mode of digestion of the SXT-Exo alkaline exonuclease**. **Panel A**: Agarose gel showing ability of SXT-Exo to digest linear dsDNA (NdeI-linerized pET28a; lanes 2-5), circularized dsDNA (undigested pET28a; lanes 6 and 7), circularized ssDNA (M13 phage DNA; lanes 8 and 9) in Tris-HCl pH7.4, 50 mM NaCl with/without 10 mM MgCl_2_; λ-HindIII (NEB) DNA ladder (lane1). **Panel B**: Agarose gel showing the ability of SXT-Exo and lambda-Exo to digest 5'-phosphorylated linear dsDNA substrates ('unmodified'; lanes 2, 3, 6 and 7), compared with analogous 5'-phosphorylated linear dsDNA substrates containing 3 consecutive phosphorothioate linkages at the 5'-termini of each strand (PT-modified; lanes 4, 5, 8 and 9). The 712 bp 'unmodified' or 'PT-modified' dsDNA substrates (0.1 mg) were incubated at 37°C with lambda-Exo (3 μg) or SXT-Exo (30 μg) in Tris-HCl, (25 mM, pH7.4), 50 mM NaCl, 10 mM MgCl_2 _(total volume 40 μl). Aliquots (20 μl) were quenched (20 mM EDTA + 1% SDS) immediately, and after 30 mins, and analyzed on 1% agarose TAE gels. 1 Kb Plus DNA Ladder (Invitrogen; lane 1). **Panel C**: Agarose gel showing time-course of digestion of 5'-phosphorylated linear dsDNA (NdeI-linearized pET28a, 0.56 pmol) by SXT-Exo (50 pmol of trimers) in Tris-HCl pH7.4, 50 mM NaCl, 10 mM MgCl_2_; at 37°C, with aliquots removed at times indicated (0-160 minutes; lanes 2-11); 1 Kb Plus DNA Ladder (lane 1).

The lambda-Exo and SPP1-Chu proteins essentially require a divalent metal ion cofactor for exonuclease activities [17,26,27]. Therefore, we first investigated the ability of SXT-Exo to digest linear dsDNA in the presence of millimolar concentrations of various divalent metal ions (Mg^2+^, Mn^2+^, Co^2+^, Cu^2+^, Ca^2+^, Zn^2+^, Ni^2+^, Fe^2+^). Results indicated that only Mn^2+ ^and Mg^2+ ^ions could function as cofactors for the dsDNA exonuclease activities of SXT-Exo (data not shown). We then investigated the substrate range of SXT-Exo, determining its ability to digest a variety of linear and (covalently-closed) circular double stranded and single stranded DNA molecules. A large excess of SXT-Exo protein was incubated with undigested pUC18 plasmid DNA to determine whether it had the ability to nick or cleave covalently closed circular dsDNA molecules (Figure 2, Panel A; lanes 6 and 7). Results clearly indicated that SXT-Exo had no endonuclease or nicking activities. Similarly, incubation with M13-phage DNA (Panel A, lanes 8 and 9) demonstrated that SXT-Exo could not cleave circularized ssDNA. In these respects, SXT-Exo has substrate specificities analogous to those of SPP1-Chu and lambda-Exo [17,21,22,26,27].

Aliquots from a time-course analysis of the SXT-Exo mediated digestion of NdeI-linearized pET28a are shown in Figure 2, Panel C. It could be seen that the concentration of linear dsDNA molecules decreased steadily over time (i.e. there was a gradual decrease in fluorescence intensity), but their sizes remained relatively unchanged. This finding was consistent with SXT-Exo digesting dsDNA with significant processivity; i.e. it sequentially hydrolyzed numerous nucleotides from each termini without dissociation of the protein from the DNA chain.

Nikiforov *et al*. have previously shown that the 5'- to 3'-exonuclease activities of the T7 gene 6 protein could be effectively inhibited by the incorporation of phoshorothioate groups into the DNA backbone, in place of 'natural' phosphodiester linkages [45]. We used an analogous approach to determine the digestion polarity of the SXT-Exo exonuclease (Figure 2, Panel B). A PCR-based strategy was used to synthesize a 5'-phosphorylated linear double stranded DNA substrate ('PT-modified', 712 bp in length) containing 3 consecutive 'nuclease resistant' phosphorothioate linkages near the 5'-termini was synthesized. An analogous 5'-phosphorylated linear dsDNA molecule without phosphorothioate modifications ('unmodified') was similarly prepared. As the phosphorothioate linkages are only present near the 5'-termini of each strand of the DNA duplex in the 'PT-modified' substrate, they should not interfere with the activities of an exonuclease with 3'- to 5'-polarity. However, an enzyme with 5'- to 3'-polarity should be significantly inhibited. Lambda-Exo and SXT-Exo were separately incubated with these two DNA substrates (37°C, 30 mins) and their digestion products were analyzed by gel electrophoresis (Figure 2, Panel B). Negligible amounts of the 'PT-modified' linear dsDNA substrate were digested by SXT-Exo under the conditions used (lane 5), whereas the 'unmodified' linear dsDNA substrate was fully digested (lane 3). Under analogous conditions, lambda-Exo completely-digested the 'unmodified' dsDNA substrate (lane 7) but only digested a small amount of the phosphorothioate modified dsDNA (lane 9). This clearly indicated that SXT-Exo digests linear dsDNA with 5'- to 3'-polarity, analogous to the lambda-Exo and SPP1-Chu proteins [17,27].

### Quantitative determination of SXT-Exo double strand DNA exonuclease activities using the sensitive fluorescent PicoGreen reagent

We quantified the dsDNA exonuclease activities of SXT-Exo under a variety of different experimental conditions using sensitive fluorescence assays incorporating the PicoGreen reagent (Invitrogen). This approach has previously been utilized by Myers and co-workers to dissect the activities of lambda-Exo and SPP1-Chu (G34.1P) [24,26,43]. The PicoGreen reagent fluoresces intensely when complexed with double stranded DNA, but exhibits very low fluorescence levels in the presence of free nucleotides or single stranded DNA chains [43]. Using this reagent, it is possible to accurately quantify the amount of dsDNA that has been enzymatically-digested. Purified PstI-linearized pUC18 DNA (2686 bp in length) was used as a representative linear dsDNA substrate for all PicoGreen assays.

**Figure 3:**
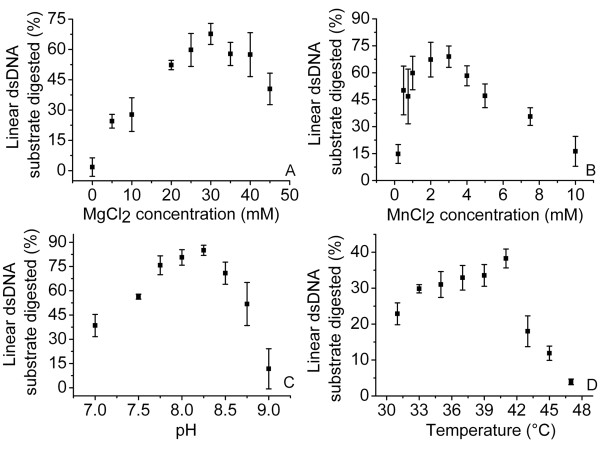
**Optimal pH, temperature and Mg(II) and Mn(II) ion concentrations for the dsDNA exonuclease activities of SXT-Exo, as determined by quenched PicoGreen Assays**. Panel** A**: Optimum Mg^2+ ^ion concentrations. SXT-Exo (2 pmol of trimers) in Tris-HCl (25 mM, pH7.4), 50 mM NaCl containing MnCl_2 _(0-10 mM); was incubated with PstI-linearized pUC18 (5 ng, 0.003 pmol) at 37°C for 30 mins. Panel **B**: Optimum Mn^2+ ^ion concentrations. SXT-Exo (2 pmol of trimers) in Tris-HCl (25 mM, pH7.4), 50 mM NaCl containing MgCl_2 _(0-50 mM); was incubated with PstI-linearized pUC18 (5 ng, 0.003 pmol) at 37°C for 30 mins. Panel** C**: Optimum pH. SXT-Exo (2 pmol of trimers) in Tris-HCl (50 mM, adjusted to pH 7.0-9.0), 50 mM NaCl, 0.5 mM MnCl_2_; was incubated with PstI-linearized pUC18 (5 ng, 0.003 pmol) at 37°C for 30 mins. Panel** D**: Optimum temperature. SXT-Exo (6 pmol of trimers) in Tris-HCl (25 mM, pH 7.4), 50 mM NaCl, 0.5 mM MnCl_2_; was incubated with PstI-linearized pUC18 (5 ng, 0.003 pmol) at 37°C for 1 min. Graphs show the the mean values ± standard deviation. See methods for detailed experimental procedures.

As our initial results had indicated that SXT-Exo essentially required Mg^2+ ^or Mn^2+ ^ions for catalytic activity, we first used quenched PicoGreen fluorescence assays to determine which concentrations these divalent metal ions were optimal for its dsDNA exonuclease activities (Figure 3). Results indicated that Mn^2+ ^was optimal at 2.5 mM (Panel B), whilst Mg^2+ ^was most effectively utilized at 30 mM (Panel A). At optimal Mg^2+ ^and Mn^2+ ^concentrations, the rates of dsDNA exonuclease activities were equivalent (within experimental error). Analogous sets of PicoGreen assays were then used to characterize the dsDNA exonuclease activities of SXT-Exo between pH 7.0 and 9.0. DNA digestion rates formed a skewed bell-shaped distribution between these pH values, with an optimum at pH 8.2 (Figure 3, Panel C). Activities fell-off markedly above pH 8.5. The operational temperature range for the SXT-Exo protein was similarly investigated. Its dsDNA exonuclease activities increased steadily between 31 and 41°C, reached a maximum at ca. 42°C, then dropped-off sharply at temperatures higher than this (Figure 3, panel D). SXT-Exo was essentially inactive at 47°C.

### Inhibition of SXT-Exo dsDNA exonuclease activities by phosphate and various inorganic salts

**Figure 4:**
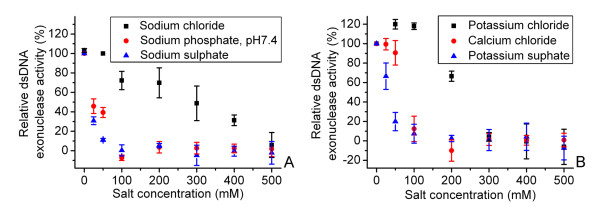
**Effects of addition of various monovalent and divalent salts on the double strand DNA activities of SXT-Exo, as determined by quenched PicoGreen assays**. Panel** A: **Inhibition of the dsDNA exonuclease activities of SXT-Exo with sodium chloride (black squares), sodium phosphate (buffered to pH7.4; red circles) and sodium sulfate (blue triangles). SXT-Exo (2 pmol of trimers), PstI-linearized pUC18 (5 ng, 0.003 pmol) in Tris-HCl (25 mM, pH7.4), 0.5 mM MnCl_2_; as well as the salt indicated in the figure (NaCl, Na_2_HPO_4 _(pH7.4), or Na_2_SO_4_; 0-500 mM); were incubated at 37°C for 30 mins. Panel** B: **Inhibition of the dsDNA exonuclease activities of SXT-Exo with potassium chloride (black squares), calcium chloride (red squares) and potassium sulfate (blue triangles). SXT-Exo (2 pmol of trimers), PstI-linearized pUC18 (5 ng, 0.003 pmol) in Tris-HCl (25 mM, pH7.4), 0.5 mM MnCl_2_; as well as the salt indicated in the figure (KCl, CaCl_2 _or K_2_SO_4_; 0-500 mM); were incubated at 37°C for 30 mins. Relative dsDNA exonuclease activities were calculated (as a percentage) by comparison with results from analogous assays that contained: SXT-Exo (2 pmol of trimers), PstI-linearized pUC18 (5 ng, 0.003 pmol) in Tris-HCl (25 mM, pH7.4), 0.5 mM MnCl_2_, 50 mM NaCl. Graphs show the the mean values ± standard deviation. See methods for detailed experimental procedures.

To gain a better understanding of other factors influencing the biochemical activities of SXT-Exo, we systematically investigated how the addition of various inorganic salts affected its ability to digest linear dsDNA (PstI-linearized pUC18). Analogous sets of quenched PicoGreen fluorescence assays were used to determine the relative effects of sodium (Na^+^), potassium (K^+^) and calcium (Ca^2+^) cations; as well as chloride (Cl^-^), phosphate (PO_4_^3-^) and sulfate (SO_4_^2-^) anions. Phosphate ions strongly inhibited the dsDNA exonuclease activities of SXT-Exo, with sodium phosphate having an IC_50 _of 25 mM (Figure 4, Panel A). This may be due to competition for the DNA internucleotide phosphodiester group binding sites within the protein trimer, or may possibly be due to interference with the putative protein binding site for the 5'-PO_4 _group at the terminus of the DNA strand being digested [24]. Electrostatic effects or divalent metal ion chelation effects (reducing the pool of free Mg^2+ ^or Mn^2+ ^ions available to the protein) may also play a role. Sulfate ions similarly inhibited SXT-Exo at concentrations above ca. 10 mM; with sodium sulfate having an IC_50 _of ca. 20 mM, and potassium sulfate having an IC_50 _of ca. 35 mM (Figure 4, Panels A and B). Its mode of inhibition may be analogous to that of phosphate, as this anion contains a roughly-similar geometrical arrangement of oxygen atoms around a central non-metal atom.

The dsDNA exonuclease activities of SXT-Exo steadily decreased with increasing concentrations of Na^+ ^and K^+ ^ions, but were only significantly inhibited at concentrations above ca. 200-300 mM (Figure 4, Panels A and B). This general inhibitory effect by Na^+ ^and K^+ ^ions at high concentration appears to be common to most, if not all related exonucleases, but the reported inhibitory sensitivities vary significantly, e.g. see references [46-51]. At the concentration used in the standard exonuclease buffer (50 mM), NaCl appeared to have negligible inhibitory or stimulatory effects. Notably however, potassium ions stimulated enzymatic activities 20% at concentrations between 50-100 mM. Baylis *et al*. [46] and Stolzenberg and Ooka [47] similarly noted that the BGL5 alkaline exonuclease from Epstein-Barr virus had optimal activities in the presence of ca. 50 mM of potassium or sodium ions. 100 mM CaCl_2 _almost entirely inhibited SXT-Exo dsDNA exonuclease activities (IC_50 _ca. 75 mM, Figure 4 Panel B). By analogy with structural observations previously noted for lambda-Exo [19], and the fact that calcium ions also inhibit the RecE [52] and RecBC exonucleases [53], it appears most likely that Ca^2+ ^inhibits SXT-Exo via direct competition with the divalent metal ion (Mg^2+ ^or Mn^2+^) binding sites.

### DNA substrate preferences

**Figure 5:**
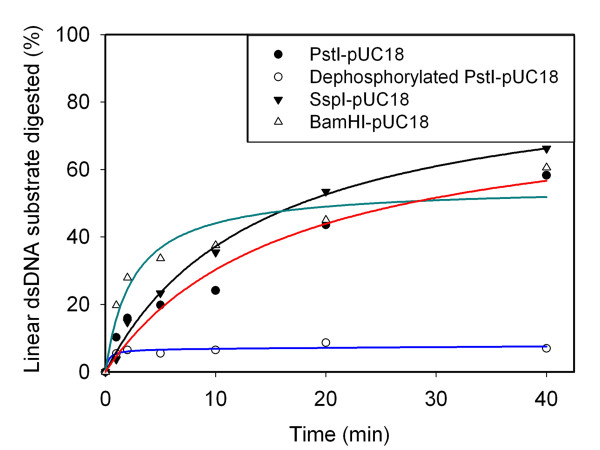
**Double stranded DNA end preferences of SXT-Exo**. SXT-Exo (2 pmol of trimers) in Tris-HCl (25 mM, pH7.4), 0.5 mM MnCl_2_, 50 mM NaCl; was incubated at 37°C for 30 mins with: i) 5'-phosphorylated linear dsDNA with 4 nt 3'-overhangs (PstI-linearized pUC18, black shaded circles); ii) 5'-hydroxylated linear dsDNA with 4 nt 3'-overhangs (dephosphorylated PstI-linerarized pUC18, un-shaded circles); iii) 5'-phosphorylated blunt-ended dsDNA (SspI-linerized pUC18, shaded inverted triangles); or iv) 5'-phosphorylated linear dsDNA with 4 nt 5'-overhangs (BamHI-linearized pUC18, un-shaded green triangles). Aliquots were removed at 1, 2, 5, 10, 20 and 40 minutes; quenched, then dsDNA levels were quantified using the PicoGreen reagent. Graphs show the the mean values ± standard deviation. See methods for detailed experimental procedures.

Having determined how various chemical and physical parameters affect the dsDNA activities of the SXT-Exo protein, we next focussed on characterizing its DNA substrate preferences in a quantitative manner. Sets of PicoGreen assays analogous to those described above were used to investigate the rates at which SXT-Exo digested linear dsDNA molecules containing different types of ends (see Figure 5). Blunt-ended linear dsDNA substrates, as well as ones containing small (4 nucleotide) 3'- or 5'-ssDNA overhangs at each terminus, were prepared by digesting pUC18 plasmid DNA with the SspI, PstI or BamHI restriction enzymes, respectively. Linear dsDNA molecules containing short 5'-ssDNA overhangs at each termini (un-shaded triangles) appeared to be digested ca. 30-50% faster during the initial phase of incubation with SXT-Exo (0-5 minutes) than corresponding blunt-ended substrates, or ones containing short 3'-overhangs (black shaded circles). However, after ca. 10 minutes this initial effect was compensated for; and after 40 minutes there were no significant differences in the overall percentages of linear dsDNA substrate consumed. Notably, SXT-Exo digested PstI-linearized pUC18 (black shaded circles) ca. 20-fold more effectively than 5'-dephosphorylated PstI-linearized pUC18 (un-shaded circles). This demonstrated that SXT-Exo had a strong preference for linear dsDNA molecules that contained 5'-phosphorylated ends, compared with ones that had lacked 5'-phosphate groups.

**Figure 6:**
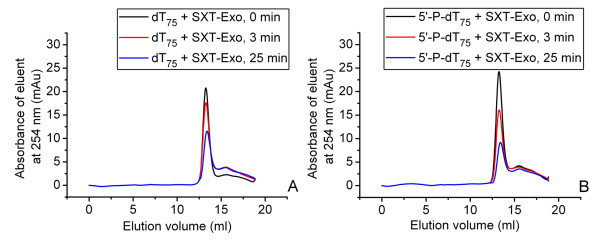
**Digestion of 5'-phosphorylated and non-phosphorylated oligonucleotides by SXT-Exo, monitored by size exclusion chromatography**. Panel **A**: Overlay of three size exclusion chromatograms of the time-wise digestion of a non-phosphorylated synthetic 75 mer of oligothymidine (dT_75_, 400 pmol) by SXT-Exo (100 pmol of trimers) in 25 mM Tris-HCl pH7.4, 50 mM NaCl, 10 mM MgCl_2_; incubated at 37°C. Black-coloured trace: amounts of oligo-dT_75 _present immediately after addition of SXT-Exo; red and blue-coloured traces: oligo-dT_75 _present 3 minutes, and 25 minutes after the addition of SXT-Exo, respectively. Panel** B**: Overlay of three size exclusion chromatograms of the time-wise digestion of a 5'-phosphorylated 75-mer of oligothymidine (5'-P-dT_75_, 400 pmol) by SXT-Exo (100 pmol of trimers) in 25 mM Tris-HCl pH7.4, 50 mM NaCl, 10 mM MgCl_2_; incubated at 37°C. Black-coloured trace: 5'-phosphorylated oligo-dT_75 _present immediately after the addition of SXT-Exo; red and blue-coloured traces: 5'-phosphorylated oligo-dT_75 _present 3 minutes, and 25 minutes after the addition of SXT-Exo, respectively.

To determine whether SXT-Exo had ssDNA 'trimming' activity [21,23] we investigated its ability to digest both non-phosphorylated and 5'-phosphorylated forms of a synthetic 75 mer of oligothymidine (dT_75 _and 5'-P-dT_75_, respectively). SXT-Exo (100 pmol of trimers) was incubated with 400 pmol of dT_75 _or 5'-P-dT_75 _in Tris-HCl buffer (pH7.4) containing 10 mM MgCl_2 _at 37°C. Reactions were quenched immediately (0 min), as well as after 3 minutes and 25 minutes; then products were analyzed by size-exclusion chromatography. This enabled both the length (retention time) and concentration (peak area) of oligonucleotide reaction products to be determined. Overlays of the three chromatograms obtained for reaction products formed at the three time points are shown in Figure 6 (Panel A, dT_75_; Panel B, 5'-P-dT_75_). Various amounts of oligo-dT_75 _(10-80 nmoles) were analogously analyzed using gel filtration chromatography, to confirm that the area under the main peak eluting at 12.9 ml on the chromatogram was proportional to the amount of oligonucleotide present (Additional File 4, Panel A). Under the conditions used, SXT-Exo digested 13% and 40% of the dT_75 _substrate after 3 minutes and 25 minutes, respectively (Panel A). In the corresponding set of reactions, 37% and 63% of the 5'-P-dT_75 _substrate was digested after 3 minutes and 25 minutes, respectively (Panel B). This revealed that SXT-Exo had a ca. 1.5- to 3-fold preference for 5'-phosphorylated ends under the conditions tested. It may be noted on both chromatograms, that the height of the shoulder peaks did not concomitantly increase as the main peak decreased, and no additional peaks with longer retention times appeared. This revealed that SXT-Exo digested both the phosphorylated and non-phosphorylated forms of the oligothymidine 75-mer without the production of significant amounts of shorter chain length products. This observation is consistent with SXT-Exo hydrolyzing ssDNA with a certain level of processivity.

**Figure 7:**
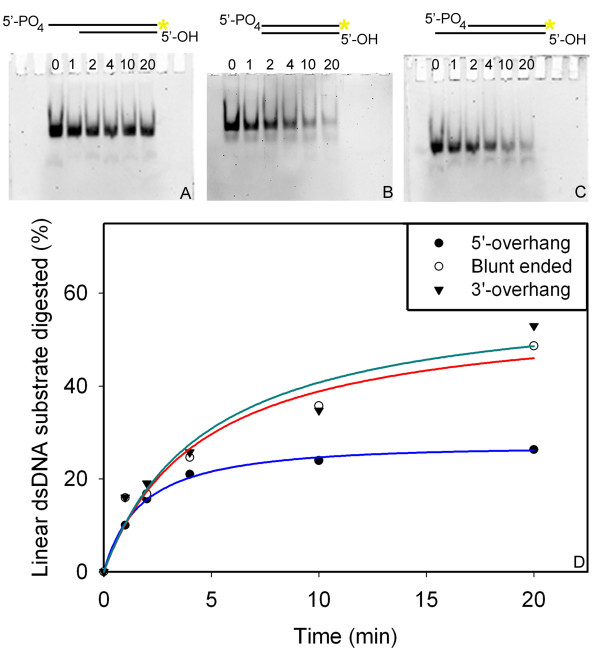
**Digestion of fluorescently-labeled annealed oligonucleotide substrates by SXT-Exo**. The ability of SXT-Exo to digest three different (partially) dsDNA substrates (prepared from two annealed oligonucleotides, see Additional File 4) was monitored by quantifying the digestion of the 5'-phosphorylated-3'-Cy3-labeled strand using fluorescence gel scanning. In each assay, SXT-Exo (50 pmol of trimers) was incubated at 25°C with 20 pmol of the dsDNA substrate in 50 mM Tris-HCl pH8.0, 0.5 mM MnCl_2_. Aliquots were removed and quenched at 0, 1, 2, 4, 10 and 20 minutes; then analyzed on 7 M urea-TBE denaturing polyacrylamide gels (times indicated above lanes). Gels were scanned for fluorescence, and fluorescence intensities of the bands corresponding to the Cy3-labeled strand were quantified. Panel** A**: Representative fluorescence-scanned gel image showing time-wise digestion of the 5'-overhang DNA substrate (annealed 5'-PO_4_-70Cy3 + 50blunt oligonucleotides) by SXT-Exo. Panel** B**: Representative gel image showing digestion of the Blunt ended DNA substrate (annealed 5'-PO_4_-50Cy3 + 50blunt oligonucleotides) by SXT-Exo. Panel** C**: Representative gel image showing digestion of the 3'-overhang DNA substrate (annealed 5'-PO_4_-50Cy3 + 70overhang oligonucleotides) by SXT-Exo. Panel** D**: Plot showing the digestion of the three DNA substrates by SXT-Exo over a 20 minute period; reported as the mean percentage ± standard deviation, based on three independent replicates. See materials section for details.

**Figure 8:**
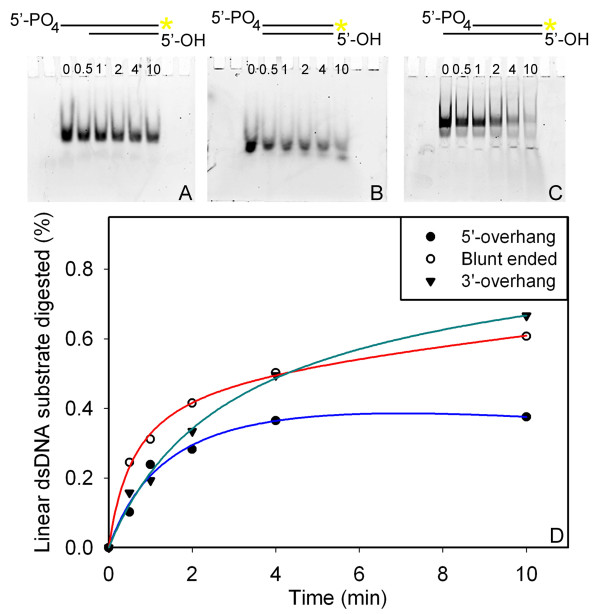
**Digestion of fluorescently-labeled annealed oligonucleotide substrates by lambda-Exo**. In experiments analogous to those described for SXT-Exo (see Figure 7), the ability of lambda-Exo to digest three different (partially) dsDNA substrates was investigated. In each assay, lambda-Exo (3 pmol of trimers) was incubated at 25°C with 20 pmol of the dsDNA substrate in 50 mM Tris-HCl pH8.0, 5 mM MgCl_2_. Aliquots were removed and quenched at 0, 0.5, 1, 2, 4 and 10 minutes; then analyzed on 7 M urea-TBE denaturing polyacrylamide gels (times indicated above lanes). Gels were scanned for fluorescence, and fluorescence intensities of the bands corresponding to the Cy3-labeled strand were quantified. Panel** A**: Representative fluorescence-scanned gel image showing time-wise digestion of the 5'-overhang DNA substrate by SXT-Exo. Panel** B**: Representative gel image showing digestion of the Blunt ended DNA substrate by lambda-Exo. Panel **C**: Representative gel image showing digestion of the 3'-overhang DNA substrate by lambda-Exo. Panel** D**: Plot showing the digestion of the three DNA substrates by lambda-Exo over a 10 minute period; reported as the mean percentage ± standard deviation, based on three independent replicates. See materials section for details.

To further dissect the dsDNA-end preferences of the SXT-Exo protein, three (partially) double stranded substrates were created by annealing (partially) complementary pairs of oligonucleotides (see Additional File 4, Panel B). One of the annealed oligonucleotides in each substrate was phosphorylated at its 5'-termini and fluorescently-labelled at its 3'-termini with the Cy3 group (indicated with an orange-coloured asterisk in Figures 7 and 8). This enabled the digestion of this DNA strand to be followed by fluorescence scanning of denaturing polyacrylamide gels (with the other strand being 'invisible'). The "Blunt ended" substrate comprised a 50 bp fully double stranded 'core' region; the "5'-overhang" substrate contained the 'core' dsDNA region and a 5'-overhang of 20 thymidine residues; and the "3'-overhang" substrate contained a 3'-overhang of 20 thymidine residues (which may also be referred to as a 5'-recessed end). The composition of our exonuclease substrates are similar to those previously used by Mitsis and Kwagh [54] for their characterization of lambda-Exo.

SXT-Exo (50 pmol of trimers) was separately incubated with 20 pmol of each of the annealed oligonucleotide substrates at 25°C for 20 minutes (Figure 7). Aliquots were periodically removed, quenched, and then resolved on denaturing-polyacrylamide gels. Gels were fluorescently-scanned, and the intensities of the bands that corresponded to the Cy3-labeled oligos were quantified to determine the percentage of substrate digested at each time point (Panel D). Four replicates were performed; and representative gel images are shown in Panels A, B and C. Standard curves were constructed for both Cy3-labelled oligos used, to confirm that their fluorescence intensities were linear over the range of concentrations used (Additional File 4, Panels C and D). Results indicated that SXT-Exo digested DNA substrates containing 5'-recessed ends (3'-overhangs; shaded inverted triangles) or blunt-ends (un-shaded circles) with roughly equal efficiencies (52% and 48% digested, respectively; see Figure 7 Panel D). Both were digested roughly 2-fold more effectively than ones containing 5'-overhangs (shaded circles, 26% digested). Furthermore, it may be noted that the intensities of the bands on the gel corresponding to the 5'-phosphorylated-3'-fluorescently labelled oligonucleotides (5'-PO_4_-70Cy3 or 5'-PO_4_-50Cy3) decreased with increasing time, but their size remained essentially constant. This indicated that this strand of the (partial) duplex was being digested by SXT-Exo in the 5'- to 3'-direction, in an apparently processive manner.

Analogous sets of experiments were performed with recombinant lambda-Exo protein (Figure 8); except using fewer molar equivalents of enzyme (3 pmol of trimers) and a shorter incubation time (10 minutes). Lambda-Exo digested the DNA substrates containing 5'-recessed ends (shaded inverted triangles) and blunt-ends (un-shaded circles) with roughly equal efficiencies (66% and 60% digested, respectively; see Figure 8 Panel D). Both of these DNA substrates were digested roughly twice as effectively as ones containing 5'-overhangs (shaded circles, 37% digested). Our findings are entirely consistent with the results reported by Mitsis and Kwagh [54]. Taken together, these experiments clearly demonstrated that the lambda-Exo and SXT-Exo proteins digested this set of (partially) dsDNA substrates in an analogous manner; with both proteins exhibiting equivalent substrate preferences.

### Processivity and rate of double strand DNA digestion by SXT-Exo

**Figure 9:**
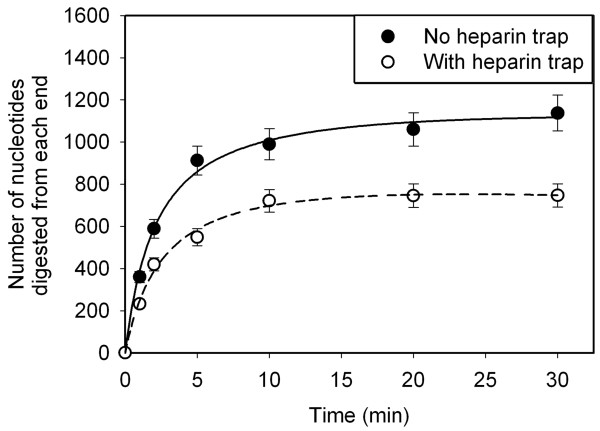
**Processivity of SXT-Exo digestion of double stranded DNA**. Heparin-trap experiments were used to calculate the average number of nucleotides hydrolyzed by an SXT-Exo trimer during a single binding event. SXT-Exo (41 nmol of trimers) and PstI-linearized pUC18 DNA (2686 bp in length; 18.1 pmol) in Tris-HCl (25 mM, pH7.4), 50 mM NaCl, 0.5 mM MnCl_2_; were incubated at 25°C for 30 s, before trapping unbound protein by addition of a large excess of heparin. Aliquots were removed at 0, 1, 2, 5, 10, 20 and 30 minutes; quenched, then dsDNA levels immediately quantified using PicoGreen assays to determine the number of nucleotides digested from each end (red circles) at each time point. Analogous control experiments without heparin were performed (black circles). Four independent replicates of each experiment were conducted, and graphs show the mean values ± standard deviation. See materials section for details.

The processivity of the SXT-Exo enzyme was determined using a 'heparin trap' method [55]. This approach was previously used by Myers and co-workers to characterize the processivity of the lambda-Exo and SPP1-Chu proteins [24,26]. A ca. 2000-fold molar excess of SXT-Exo trimers was incubated with the linear double stranded DNA substrate (PstI-linearized pUC18 with 5'-phosphorylated ends), to ensure that both termini were saturated. After allowing 30 seconds for initiation of reaction, a large excess of heparin was added to sequester all unbound enzyme, to ensure that we were monitoring 'single-binding' digestion events; i.e. determining the average number of nucleotides digested by an 'active' SXT-Exo trimer before dissociation. Under the conditions employed (Tris-HCl pH 7.4, 0.5 mM MnCl_2_, 25°C), the processivity of SXT-Exo was estimated to be 746 ± 55 nucleotides (un-shaded circles, Figure 9). Analogous control experiments were performed (no heparin trap) to quantify the DNA digested by equivalent amounts of SXT-Exo during multiple protein binding events; i.e. including further digestion of partially-digested DNA substrates by re-associated SXT-Exo trimers. Under these conditions 1137 ± 85 nucleotides were digested in 30 minutes (shaded circles, Figure 9). As it accurately reflects the digestion of dsDNA molecules (from each terminus) by equimolar amounts of SXT-Exo protein trimers, the heparin trap data was also used to determine the initial (maximal) rate of digestion. Fitting the data to a hyperbola [24], the rate of SXT-Exo mediated digestion of PstI-linearized pUC18 DNA was gauged to be ca. 7 nt per second.

### Protein-mediated modulation of the double stranded DNA exonuclease activities of SXT-Exo and lambda-Exo

**Figure 10:**
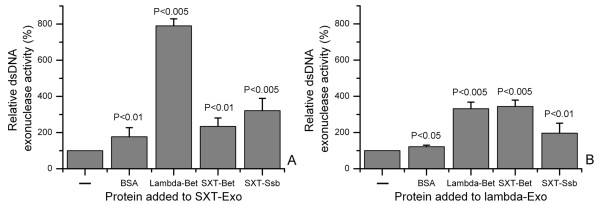
**Stimulation of double strand DNA exonuclease activities of SXT-Exo and lambda-Exo by SSAP and Ssb proteins**. Panel** A**. SXT-Exo (2 pmol of trimers), PstI-linearized pUC18 (5 ng, 0.003 pmol) and 2 pmol of the protein indicated in the text (BSA, lambda-Bet, SXT-Bet or SXT-Ssb) in Tris-HCl (25 mM, pH7.4), 50 mM NaCl, 0.5 mM MnCl_2_; were incubated at 25°C for 30 mins before EDTA quenching. dsDNA levels were immediately quantified using PicoGreen reagent. The level of DNA digestion by SXT-Exo in the absence of added protein (-) was normalized to a value of 100%. Panel **B**. In analogous sets of experiments, lambda-Exo (2 pmol of trimers), PstI-linearized pUC18 (5 ng, 0.003 pmol) and 2 pmol of BSA, lambda-Bet, SXT-Bet or SXT-Ssb; in Tris-HCl (25 mM, pH7.4), 50 mM NaCl, 5 mM MgCl_2_; were incubated at 25°C for 10 mins. Digestion levels were normalized to those of lambda-Exo in the absence of added protein (-). See methods section for detailed experimental procedure. Six independent replicates were performed for each experiment, and error bars indicate standard deviation from the mean values. Analysis using ANOVA indicated all results were statistically significant (P < 0.05) when compared to the no-protein control (-), with respective P values indicated above each bar.

Further sets of quenched PicoGreen assays (analogous to those described above) were used to determine whether the presence of equimolar amounts of the SXT-Bet or SXT-Ssb proteins had any effects on the rate by which SXT-Exo could digest a linear dsDNA molecule (PstI-linearized pUC18). Bovine serum albumin (BSA) was included as a control, as it is commonly found to enhance protein stability in solution (e.g. restriction enzymes [56]), but would not be predicted to exhibit any protein-specific effects with SXT-Exo. As may be seen in Figure 10, Panel A, the presence of SXT-Bet or SXT-Ssb enhanced the activities of SXT-Exo ca. 2.2-fold or 3.2-fold, respectively. This may be compared to a ca. 1.8-fold increase of dsDNA exonuclease activities when equimolar amounts of BSA were added. Intriguingly, the addition of lambda-Bet protein stimulated the activities of SXT-Exo nearly 8-fold. Analogous experiments were performed using the lambda-Exo protein, in place of SXT-Exo (see Figure 10, panel B). Similar results were obtained. BSA stimulated the activities of lambda-Exo ca. 1.2-fold, whilst lambda-Bet stimulated its activities ca. 3.3-fold. SXT-Bet and SXT-Ssb enhanced the dsDNA exonuclease activities of lambda-Exo ca. 3.4-fold and 1.9-fold, respectively. The effects of adding different molar ratios of SSAP or Ssb proteins to SXT-Exo or lambda-Exo were not determined.

### DNA recombination activities of SXT-Bet and SXT-Exo

**Figure 11:**
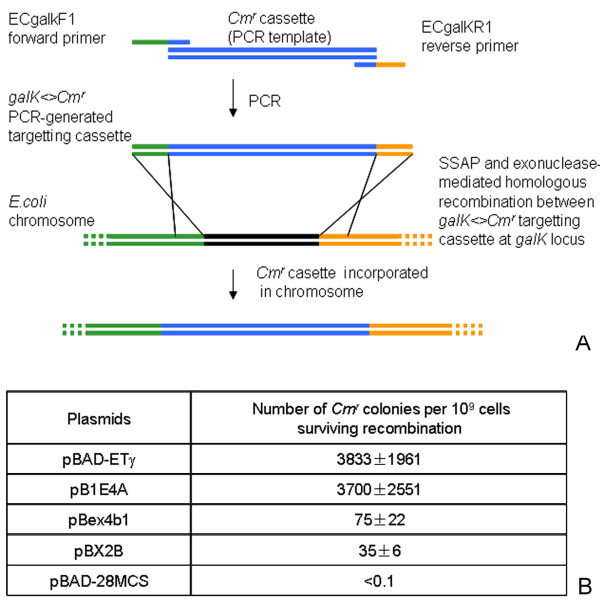
**Efficiency of SXT-Bet + SXT-Exo mediated recombination between a PCR-generated DNA fragment and its homologous target on the *E. coli *chromosome**. Panel** A: **Schematic overview of the chromosomal targeting assay use to score exonuclease + SSAP-mediate recombination efficiency. A dsDNA 'targeting' molecule (*galK<>Cm^r^*) was synthesized by PCR using two primers sharing (ca. 20 nt) sequence homology to a chloramphenicol resistance cassette at one end; and 50 nt of sequence homology to the 5'-end (ECgalKF1) or 3'-end (ECgalKR1) of the non-essential *E. coli galK *gene at the other. The purified *galK<>Cm^r ^*targeting cassette was electroporated into DH10B cells expressing pairs of Exo and SSAP proteins from established arabinose-inducible plasmids. The Exo and SSAP proteins mediate homologous recombination between the *galK<>Cm^r ^*dsDNA cassette and the *galK *locus of the *E. coli *chromosome via 50 bp regions of flanking sequence homology. This creates a mutant *E. coli *strain that has its *galK *gene replaced with a chloramphencol resistance cassette. Panel** B**. Comparison of dsDNA recombination efficiencies of SXT-Bet-Exo with those of RecET and lambda-Bet-Exo. The RecET; lambda-Bet-Exo; SXT-Bet-Exo and SXT-Ssb-Bet-Exo sets of homologous recombination-promoting proteins were expressed from arabinose-inducible (P_BAD_/araC) plasmids established in *E. coli *DH10B cells (see Additional File 3). pBAD-ETγ: RecE + RecT + lambda-Gam [35]; pB1E4A: lambda-Bet + lambda Exo; pBex4b1: SXT-Bet + SXT-Exo; pBX2B: SXT-Ssb + SXT-Bet + SXT-Exo; pBAD-28MCS (negative control). Homologous recombination events were scored by plating *galK<>Cm^r^*-electroporated cells onto LB-agar with/without chloramphenicol. The dsDNA recombination efficiency was calculated by dividing the number of Cm^r ^colonies by the total number of cells (CFUs) that survived electroporation. 8 replicates were performed; the mean recombination efficiency ± standard deviation is reported.

In order to evaluate whether the SXT-Bet and Exo proteins had the combined ability to promote homologous DNA recombination, a non-essential gene on the *E. coli *chromosome (*galK*) was targeted for allelic replacement with a PCR-generated dsDNA chloramphenicol resistance cassette (*galK<>Cm^r^*). An analogous approach was previously used by Yu *et al*. [38], to determine the dsDNA recombination activities of the lambda-Gam-Bet-Exo proteins (the lambda-Red system), expressed from a modified prophage element in the DY380 strain of *E. coli*. At its 5'-end, the *galK<>Cm^r ^*targeting cassette contains 50 bp of sequence homologous to the chromosomal region immediately upstream of the *galK *gene, and 50 bp of sequence at its 3'-end homologous to a region in the *galK *gene that is slightly upstream of the termination codon (see Figure 11, Panel A).

The *lambda-bet-exo*, *SXT-bet-exo *and *SXT-ssb-bet-exo *genes were analogously expressed from arabinose-inducible plasmids (pB1E4, pBex4b1 and pBX2B, respectively) constructed from pG5P4-1 which contains the 'backbone' of the pBAD-ETγ plasmid [35] (see Additional File 3). An analogous arabinose-inducible plasmid (pBAD-28MCS) containing the multiple cloning site from pET28a in place of a gene encoding a homologous recombination protein was used as a negative control, and pBAD-ETγ was included as an additional positive control. pBAD-ETγ contains a gene encoding the functional C-terminal domain of the RecE protein under the control of the arabinose-inducible P_BAD _promoter, as well as its partnering *recT *gene constitutively expressed from a synthetic EM7 promoter and the *lambda-gam *gene constitutively expressed from a TN5 promoter. Plasmids were stably established within the DH10B strain of *E. coli*, which was selected due to its high electroporation efficiency, and because it does not express the RecA recombinase. Protein expression was induced by the addition of arabinose to 0.1%, and transformed strains were incubated at 37°C for 1 hour to allow sufficient time for the cellular synthesis of the DNA recombination proteins, before being made competent for electroporation ('recombination competent' cells). The gene targeting efficiency was calculated by dividing the number of chloramphenicol resistant colonies by the total number of cells surviving electroporation (colony forming units). Under the conditions tested, lambda-Bet-Exo (B1E4) and RecET (pBAD-ETγ) were found to have approximately equal dsDNA homologous recombination efficiencies (3.8×10^-6 ^and 3.7×10^-6^, respectively; Figure 11, Panel B). The background recombination levels (<1 × 10^-10^, pBAD-28MCS) were extremely low, as DH10B is a *recA- *strain. The pBex4b1 (SXT-Bet-Exo) and pBX2B (SXT-Ssb-Bet-Exo) plasmids had more than 50-fold lower recombination efficiencies of 7.5×10^-8 ^and 3.5×10^-8^, respectively. However, this was still more than 300-fold higher than background levels, clearly demonstrating that the SXT-Bet and Exo proteins functioned together to promote dsDNA recombination in *E. coli*.

## Discussion

### Sequence similarities between SXT-Exo and related alkaline exonucleases of viral/phage origin

Lambda-Exo, G34.1P (SPP1-Chu) and SXT-Exo all belong to the lambda Exonuclease (LE) superfamily of alkaline exonucleases [13], which is also (somewhat confusingly) referred to as the 'YqaJ-like viral recombinase domain' (pfam09588). Genes encoding lambda-Exo/YqaJ homologues are commonly found in the genomes of prokaryotes and prokaryotic mobile genetic elements; often adjacent or proximal to genes encoding single stand DNA annealing proteins (SSAPs) and single strand DNA binding proteins (Ssbs) [12]. They fall within the large PD-(D/E)XK restriction endonuclease superfamily [15], and are phylogenetically and functionally related to alkaline exonucleases from the baculoviridae and herpesviridae families such as: UL12 from Herpes Simplex Virus type 1 (HSV-1) [47,48]; Kaposi's Sarcoma-associated Herpesvirus (KSHV) shut-off and exonuclease (SOX) protein [57]; the Epstein-Barr Virus (EBV) BGLF5 exonuclease [46,47,58]; and the alkaline nuclease (Orf133) from the *Autographa californica *multiple-capsid nucleopolyhedrovirus (AcMNPV) [50,59]. The RecE exonuclease from *E. coli *has minimal sequence homology to lambda exonuclease/YqaJ family proteins, even though it has many functional and structural similarities [51,52,60,61].

SXT-Exo (338aa) and SPP1-Chu (311aa) are notably longer than lambda-Exo (226aa); with shared homology, conserved motifs and putative active site residues located within the N-terminal 200aa region [13,26] (see Additional File 1). Lambda-Exo crystallizes as a 'doughnut-shaped' torroidal trimer [19], but recombinant G34.1P (SPP1-Chu) has been reported to form a dimer in one study [27] and a pentamer or hexamer in another [26]. Contrastingly, most eukaryotic viral alkaline exonucleases are monomeric and contain additional domains at the N- and C-termini that are involved in protein-protein interactions and DNA binding [15,57,58]. Our size exclusion chromatography data (Figure 1) strongly suggests that the active form of SXT-Exo is a torroidal trimer that is structurally and functionally analogous to lambda-Exo; even though it also shares high levels of sequence homology with SPP1 G34.1 (Chu), which appears to adopt a quite different multimeric arrangement.

### Optimal conditions for double strand DNA exonuclease activities of the SXT-Exo protein

The SXT-Exo protein had maximal dsDNA exonuclease activities in Tris-HCl buffer at a pH of 8.2 (Figure 3, Panel C), which is slightly lower, but comparable to, the pH optima of the lambda-Exo (pH 9.2-9.5 [18]) and SPP1-Chu (pH 9.0 [26]) alkaline exonucleases. Whilst lambda-Exo and SPP1-Chu optimally require Mg^2+ ^ions for catalytic activities [17,19,26,27], SXT-Exo is notably different in its preference for Mn^2+ ^ions over Mg^2+ ^ions (Figure 3, Panels A and B). Apart from the increased apparent binding affinity for Mn^2+ ^ions, results from our *in vitro *experiments (those described here, as well as those not shown) indicate that there are no distinguishable differences in substrate preferences or biochemical activities of SXT-Exo in the presence of 10 mMgCl_2 _or 0.5 mM MnCl_2_; suggesting that these two ions are functionally interchangeable. In lambda-Exo, the Asp119, Glu129 and Leu130 residues present within motifs 3 and 4 (see Additional File 1, Panel B; motif numbering according to Vellani and Myers [26]) are involved in binding the divalent metal ion putatively involved in phosphodiester bond hydrolysis [19]. These residues are entirely conserved within SXT-Exo; equating to Asp100, Glu110 and Leu111; which implies that the catalytic Mn^2+ ^or Mg^2+ ^ions are bound in an analogous manner. To the best of our knowledge, intracellular manganese and magnesium concentrations within *V. cholerae *cells have not yet been determined. However, it appears likely that the majority of SXT-Exo protein expressed within SXT-infected *V. cholerae *cells would contain Mg^2+ ^ions, due to its presumably far-higher intracellular concentrations.

### Substrate Preferences and mode of digestion

The inability of SXT-Exo to digest dsDNA molecules that contained phosphorothioate modifications near their 5'-termini, clearly indicated that this protein functions as an exonuclease with strict 5' to 3'-polarity. This is mechanistically-consistent with the finding that the 5'-phosphorylation status of DNA substrates strongly affects the efficiency by which they are enzymatically-degraded. SXT-Exo digested 5'-phosphorylated dsDNA ca. 20-fold more effectively (Figure 5), and 5'-phosphorylated ssDNA 1.5- to 3-fold more effectively (Figure 6) than the corresponding dephosphorylated substrates. It has previously been shown that lambda-Exo and G34.1P (SPP1-Chu) both exhibit similarly-strong preferences for ssDNA and dsDNA substrates with 5'-phosphorylated ends [17,24,27]. Subramanian *et al*. revealed that the Arg28 residue of lambda-Exo plays a key role in binding 5'-phosphorylated DNA substrates; with the R28A mutant exhibiting an impaired ability to 'recognize' dsDNA ends, resulting in far lower levels of exonuclease processvity compared with the wild type enzyme [24]. This arginine residue is highly conserved within this class of alkaline exonucleases [13,15]; corresponding to Arg17 in SXT-Exo and Arg20 in SPP1-Chu [26] (located within motif 1 in Additional File 1, Panel B). The notable inhibitory effects of phosphate and sulfate ions (Figure 4) are consistent with them competing for the protein binding site that accommodates the terminal 5'-phosphate group of DNA substrates.

Exonucleases may be broadly divided into two functional classes: distributive enzymes that dissociate after removal of a single nucleotide; or processive enzymes that remain bound to the DNA chain for a large number catalytic events, or until complete hydrolysis has occurred [25]. The heparin-trap experiments revealed that SXT-Exo digested dsDNA in a highly processive manner; hydrolyzing an average of 746 ± 55 nucleotides from each terminus before dissociation (Figure 9). Its rate of DNA hydrolysis was determined to be ca. 7 nt/s. The processivity and rate of digestion for SXT-Exo reported here are comparable to those previously determined for lambda-Exo using bulk scale, solution-based assays: i) a digestion rate of ca. 17 nt/s, with a processivity of ca. 400 nt [24]; ii) a digestion rate of 4-7 nt/s [54]; iii) a processivity of >3000 nt [22]. They are also similar to the figures obtained for the SPP1-Chu exonuclease: a digestion rate of 2-3 nt/s, with a processivity of ca. 1000 nt [26]. This is consistent with these three alkaline exonuclease proteins sharing a common hydrolytic mechanism.

When relatively long linear dsDNA substrates (linearized pUC18, 2686 bp) were used as substrates for the SXT-Exo protein, the presence of blunt-ends, or short (4 nt) 3'- or 5'-overhangs had little overall influence on the levels of digestion (Figure 5). However, when shorter (annealed oligonucleotide) substrates were used, which contained considerably longer (20 nt) 3'- or 5'-oligothymidine overhangs; there were notable differences in its relative digestion efficiencies (Figure 7). This suggests that below a certain threshold, the length or type of overhang present at the DNA terminus does not significantly affect the binding or 'productive loading' of the exonuclease protein; only the 5'-phosphorylation status is important. As may be seen in Figures 7 and 8, SXT-Exo and lambda-Exo exhibited an analogous ca. 2-fold preference for the digestion of 5'-recessed and blunt-ended DNA substrates, over ones containing 5'-overhangs. This is fully consistent with the results of Mitsis and Kwagh, who demonstrated that the rate constants for DNA digestion mediated by lambda-Exo decreased in the order: 10 nt 5'-recessed ends > blunt ends >>10 nt 5'-overhangs [54]. Taken together, our results indicate that the SXT-Exo and lambda-Exo proteins both process linear dsDNA ends in a similar manner, producing the long 3'-ssDNA tails that are the substrates for their respective SSAP partners.

### Stimulation of SXT-Exo activities by SSAP and Ssb proteins

It was previously shown that the G35P single strand annealing protein from bacteriophage SPP1 stimulated the exonuclease activities of its partnering G34.1P (Chu) protein towards dsDNA substrates ca. 2- to 5-fold [27]. The authors further demonstrated that the G36P single strand binding protein protected ssDNA from digestion by G34.1P. However, they did not investigate whether G36P modulated the dsDNA exonucleolytic activities of G34.1P. Here, we found that the SXT-Bet, SXT-Ssb, lambda-Bet and BSA proteins enhanced the dsDNA exonuclease activities of both SXT-Exo and lambda-Exo to differing extents; and none were inhibitory. Most notably, the lambda-Bet SSAP protein stimulated the activities of SXT-Exo almost 8-fold, which was substantially higher than the 2.2-fold stimulation in the presence of SXT-Bet (see Figure 10, Panel A). In the reciprocal set of experiments, SXT-Bet and lambda-Bet stimulated the dsDNA exonuclease activities of lambda-Exo to a similar extent (3.4-fold and 3.3-fold, respectively; see Figure 10, Panel B). The SXT-Ssb protein stimulated the activities of SXT-Exo 3.2-fold, which was considerably higher than its 1.9-fold stimulation of lambda-Exo. There appears to be no obvious pattern or trend for the abilities of the SSAP and Ssb proteins to enhance the activities of the lambda-Exo or SXT-Exo exonucleases. Furthermore, many mechanisms appear plausible: e.g. the SSAP or Ssb proteins may open up regions of ssDNA at the termini of the linear dsDNA molecule, thereby facilitating the binding or 'loading' of the torroid-shaped exonuclease trimers. The SSAP or Ssb proteins may also promote the removal of nascent hairpins, stem-loops or other intra- or inter-strand annealed structures within the partially ssDNA molecules produced after an initial digestion event; thereby facilitating exonuclease binding and subsequent digestion. More importantly, it remains to be seen whether these *in vitro *effects have any direct correlation with the biological activities of the Ssb, Bet and Exo proteins from the SXT, lambda or related viral/phage DNA recombination systems; either in their native arrangements, or in alternative permutations.

### SXT-Exo and SXT-Bet promote DNA homologous recombination in E. coli cells

We chose to investigate the homologous recombination-promoting activities of SXT-Bet/Exo using a plasmid-based system in *E. coli*, as this would enable direct comparison with the well-studied lambda-Bet/Exo and RecET protein pairs. Furthermore, as the adjacent *SXT-ssb*, *bet *and *exo *genes have an operon-like arrangement on the SXT genetic element [2,10], this system presented a straightforward opportunity to investigate the possible involvement of the single strand DNA binding protein (SXT-Ssb, S064) protein in the dsDNA recombination process. Our results indicated that the SXT-Bet + SXT-Exo proteins (on plasmid pBex4b1) had modest dsDNA recombination activity within *E. coli*; ca. 50-fold less than those of RecET (pBAD-ETγ) or lambda-Bet/Exo (pB1E4A) under the conditions tested (Figure 11). Even though the SXT-Ssb protein stimulated the exonuclease activities of SXT-Exo *in vitro*, the additional supply of the SXT-Ssb protein (on plasmid pBX2B) did not enhance the recombination activities of SXT-Bet + SXT-Exo; it slightly reduced them.

Datta *et al*. previously reported that the oligonucleotide-directed DNA recombination activities of the SXT-Bet protein were comparable to those of lambda-Bet and RecT [39] in *E. coli*. Taken together with our findings, this suggests that the activities or biophysical properties of SXT-Exo may be limiting the combined dsDNA recombination activities of the SX-Bet/Exo protein pair. It is possible that the intracellular environment of *E. coli *may not be conducive to optimal SXT-Exo activities, whilst SXT-Bet can function quite efficiently. This may be related to the fact that SXT-Bet shares significantly higher levels of amino acid identity with lambda-Bet (55%) than SXT-Exo shares with lambda-Exo (26%; see Additional File 1). *E. coli *is the native host for the RecET and lambda-Bet/Exo proteins, but the SXT/R391 family of ICEs infect entirely different species of gamma-proteobacteria (e.g. *V. cholerae *and *P. rettgeri*). Consequently, they may be optimized for activity within quite different bacterial hosts. Interestingly, Datta *et al*. [39] further reported that all 4 pairs of SSAP and Exo proteins tested in *E. coli *[G35P + G34.1P (Chu) from bacteriophage SPP1; Orf47 + Orf48 from *Listeria monocytogenes*; OrfC + OrfB from *Legionella pneumophila *and Plu2935 + Plu2936 from *Photorhabdus luminescens*] had dsDNA recombination activities that were >1000-fold less than those of lambda-Bet/Exo (RecET was not tested). However the OrfC, Plu2935 and Orf48 SSAP proteins all had ssDNA recombination activities comparable to those of RecT or lambda-Bet. This suggests that for 3 out of 4 sets of proteins tested, the exonuclease protein may the cause of the low dsDNA recombination activities.

Yamamoto *et al*. recently reported that the lambda-Bet/Exo proteins (expressed from the pKD46 arabinose-inducible plasmid [37]) were capable of mediating homologous recombination between PCR-generated dsDNA fragments and the *V. cholerae *chromosome [62]. Their results indicated that the recombination efficiencies of lambda-Bet/Exo in *V. cholerae *cells were more than 100-fold less than in *E. coli *[39] and required 100 nt or longer flanking regions of homology on the dsDNA 'targeting cassettes'. These reduced efficiencies may be due to degradation of the introduced PCR fragments by host cell nucleases [63], or may reflect the reduced functional activities of the lambda-Bet/Exo proteins within *V. cholerae*. It would be extremely interesting to investigate the efficiency of the SXT-Bet + SXT-Exo proteins using similar approaches in *V. cholerae *cells; especially within nuclease-deficient strains more amenable to (natural or chitin-induced) DNA transformation [63], to see if these systems were better suited for recombineering approaches in this species.

### Biological and mechanistic implications of our findings

Taken together with previous findings, our results are consistent with the following mode of activity (see Additional File 2). A trimer of SXT-Exo proteins bind to the terminus of a linear dsDNA molecule: e.g. a 'broken' ICE molecule or host chromosomal double strand break (DSB), in a process that is facilitated by the presence of a 5'-phosphate group [24]. In the presence of Mn^2+ ^or Mg^2+ ^ions, the SXT-Exo trimer then digests one DNA strand in a highly processive manner from its 5'-terminus towards its 3'-end, whilst translocating along the complementary 3'-strand like a 'bead on a string' [19]. The long 3'-ssDNA overhangs formed are coated by the partnering SXT-Bet protein, forming helical nucleoprotein filaments [28,29]. These nucleofilaments associate with regions of homologous dsDNA on the host chromosome or viral genetic element, and mediate strand annealing, strand exchange and possibly strand invasion [31,32,34]. As SXT-Exo has no detectable nicking or endonuclease activities, the fully circularized double stranded or single stranded extrachromosomal forms of SXT (or related ICE) will not act as substrates in the absence of existing DNA breaks, or without the actions of host restriction endonucleases [10]. The ssDNA exonuclease activities of SXT-Exo may play a role in DNA 'strand assimilation', by 'trimming off' redundant ssDNA strands formed after the formation of joint DNA intermediates [21]. Furthermore, our finding that recombinant SXT-Exo cannot cleave circularized ssDNA indicates that it does not modify the circular single stranded form of SXT DNA that is the substrate for conjugative transfer from donor to recipient cells [3,4,10].

## Conclusions

The S066 SXT-Exo protein has both single strand and double strand DNA exonuclease activities, but no detectable endonuclease or nicking activities. Adopting a stable trimeric arrangement in solution, the dsDNA exonuclease activities of SXT-Exo are optimal at pH8.2 in the presence of 2.5 mM Mn^2+ ^ions; and are substantially enhanced in the presence of SSAP and Ssb proteins. Analogous to lambda-Exo, SXT-Exo degrades linear dsDNA with 5'- to 3'-polarity, digesting substrates containing 5'-phosphate groups with high processivity. The SXT-Exo and SXT-Bet (S065) proteins functioned together to promote homologous recombination events in *E. coli *cells, with an efficiency that was ca. 50-fold lower than that of lambda-Bet/Exo or RecET under the conditions tested.

## Authors' contributions

JWSH, JDH and RMW conceived of the study, and designed and coordinated the experimental work. WYC and RMW performed all of the experimental work. WYC, JWSH, JDH and RMW analyzed experimental data, and drafted the manuscript; with the final version written by RMW. All authors read and approved the final manuscript.

## Authors' information

WYC undertook his PhD at the Chinese University of Hong Kong under the supervision of JWSH. WYC now works as a Postdoctoral Fellow at the University of Hong Kong under the supervision of RMW. RMW and JDH have collaborated over the past eight years on projects concerned with the characterization and utilization of bacterial DNA recombination proteins.

## Supplementary Material

Additional file 1 **Arrangement of the *exo*, *bet *and *ssb *genes on the SXT genetic element, and alignment of the SXT-Exo protein sequence with selected alkaline exonucleases of viral/phage origin**. Panel** A**. The SXT-Exo and lambda-Exo proteins share 26% amino acid identity within a conserved ca. 200 amino acid N-terminal domain. The SXT-Bet and lambda-Bet proteins share 55% amino acid identity within a conserved ca. 200 amino acid N-terminal domain. Panel** B**. Alignment of the SXT-Exo protein sequence with those of lambda-Exo, SPP1-Chu (G34.1P) and gp47 from Listeria phage A118 (performed using Clustal). Conserved motifs and structural elements observed in the crystal structure of lambda-Exo (PDB code 1AVQ) are indicated. Click here for file

Additional file 2 **Schematic overview of Exo and SSAP-mediated recombination between two linear double stranded DNA molecules sharing sequence homology near their respective termini**. One strand from each of the two linear DNA molecules is digested in a processive manner from its 5'-terminus by the alkaline exonuclease protein (e.g. SXT-Exo), generating long 3'-single stranded DNA (ssDNA) 'tails'. The partnering single strand annealing protein (SSAP; e.g. SXT-Bet) coats these long ssDNA tails, forming helical protein nucleofilaments. The SSAP mediates annealing of the 3'-ssDNA-nucleofilament tail with a complementary region of ssDNA on the other resected DNA molecule (here, also depicted as being coated with SSAP, although this may or may not be the case). Here, the torroidal trimer of Exo protein is shown to dissociate from the non-digested strand, although this may not necessarily be the case. The SSAP mediates displacement of the original complementary strand, and promotes annealing of the (SSAP-coated) complementary strand from the other DNA molecule until its 3'-terminus, or until the end of its sequence homology. Any 3'-ssDNA overhangs would not be digested by Exo, but by a host exonuclease with 3'-5' ssDNA exonuclease activity (not shown). The Exo protein 'trims' both the 5'-ssDNA overhangs via its ssDNA exonucleolytic activity, until only a nick remains, which is sealed by host DNA ligase. Click here for file

Additional file 3 **Maps for the plasmids used in this study**. Click here for file

Additional file 4 **Chromatograms of a range of concentrations of the dT_75 _oligonucleotide; fluorescence-scanned gel images of the 5'-phosphoryrated 50Cy3 and 70Cy3 oligonucleotides, and composition of the annealed oligonucleotide substrates used to characterize exonuclease activities**. **Panel A**. Overlaid gel filtration chromatograms obtained for various concentrations (10-80 nmol) of the dT_75 _oligonucleotide used in the single strand exonuclease assays. Conditions used were identical to those described in the materials and methods section. **Panel B**: Composition of the 5'-overhang, Blunt ended and 3'-overhang substrates used to characterize the exonuclease activities of the SXT-Exo and lambda-Exo proteins. **Panel C**: fluorescence-scanned image of various concentrations of the 5'-phosphorylated-50Cy3 oligonucleotide (8 - 0.0625 pmol) resolved on a 7 M urea-TBE denaturing gel. **Panel D**: fluorescence-scanned image of various concentrations of the 5'-phosphorylated-70Cy3 oligonucleotide (8 - 0.0625 pmol) resolved on a 7 M urea-TBE denaturing gel. The band at the base of each lane corresponds to the dye used in the loading buffer. Click here for file
