# Supplementary figures and images for: Functional characterization of an alkaline exonuclease and single strand annealing protein from the SXT genetic element of Vibrio cholerae
Source: BMC Mol Biol. 2011 Apr 18;12:16. doi: 10.1186/1471-2199-12-16 (PMC3118119; doi:10.1186/1471-2199-12-16)

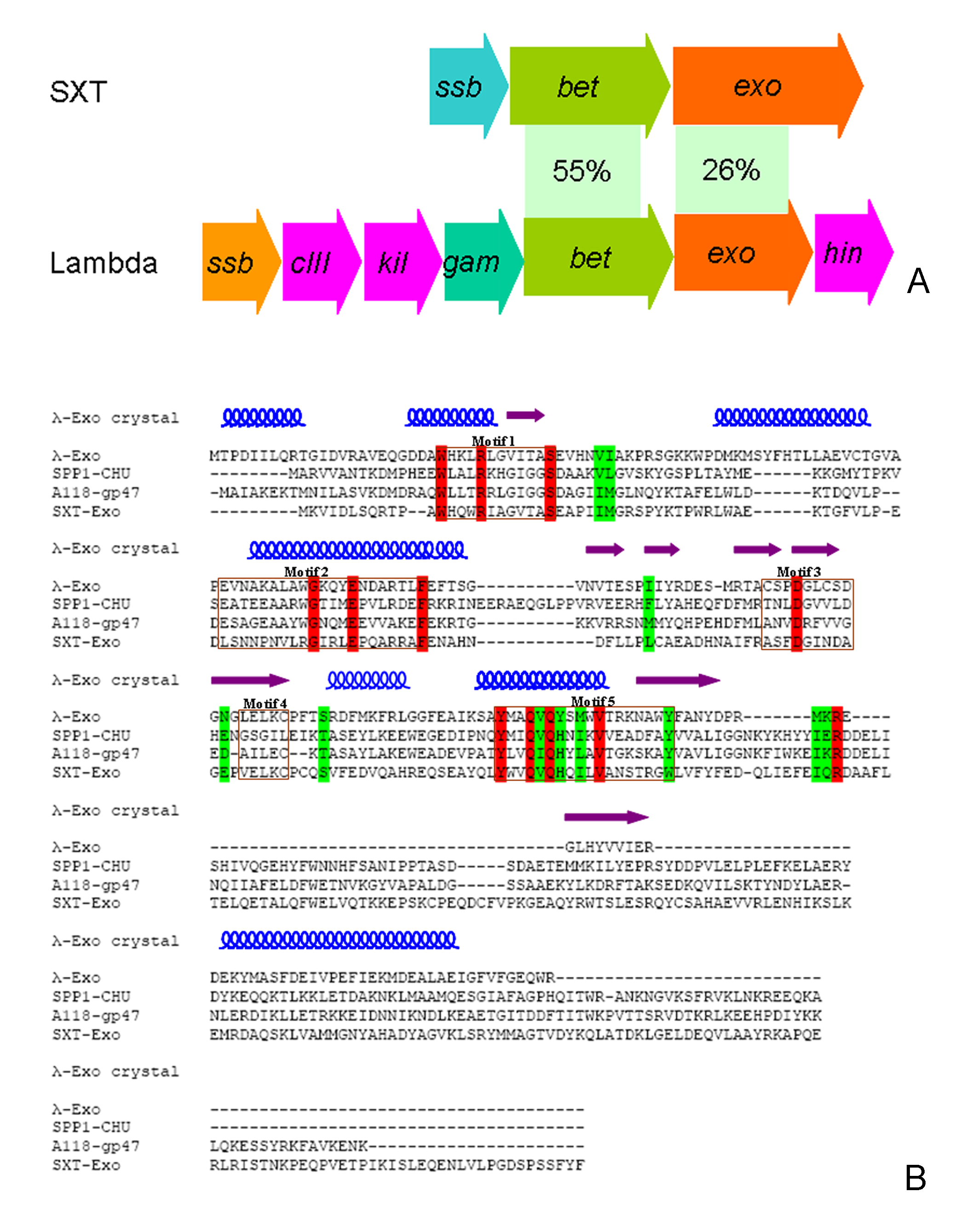

Supplement: Additional file 1 — Arrangement of the exo, bet and ssb genes on the SXT genetic element, and alignment of the SXT-Exo protein sequence with selected alkaline exonucleases of viral/phage origin. Panel A. The SXT-Exo and lambda-Exo proteins share 26% amino acid identity within a conserved ca. 200 amino acid N-terminal domain. The SXT-Bet and lambda-Bet proteins share 55% amino acid identity within a conserved ca. 200 amino acid N-terminal domain. Panel B. Alignment of the SXT-Exo protein sequence with those of lambda-Exo, SPP1-Chu (G34.1P) and gp47 from Listeria phage A118 (performed using Clustal). Conserved motifs and structural elements observed in the crystal structure of lambda-Exo (PDB code 1AVQ) are indicated. [file 1471-2199-12-16-S1.JPEG]

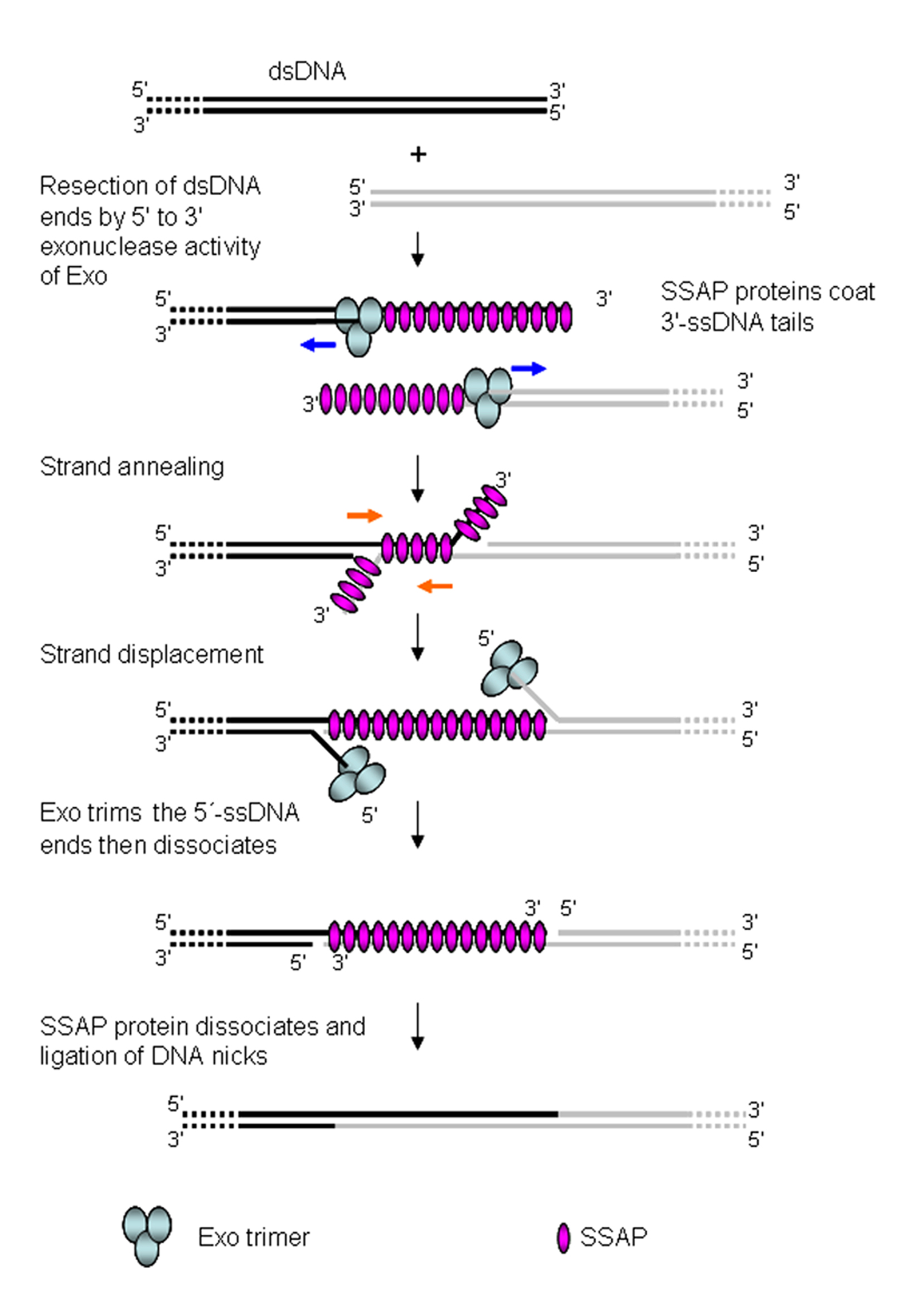

Supplement: Additional file 2 — Schematic overview of Exo and SSAP-mediated recombination between two linear double stranded DNA molecules sharing sequence homology near their respective termini. One strand from each of the two linear DNA molecules is digested in a processive manner from its 5'-terminus by the alkaline exonuclease protein (e.g. SXT-Exo), generating long 3'-single stranded DNA (ssDNA) 'tails'. The partnering single strand annealing protein (SSAP; e.g. SXT-Bet) coats these long ssDNA tails, forming helical protein nucleofilaments. The SSAP mediates annealing of the 3'-ssDNA-nucleofilament tail with a complementary region of ssDNA on the other resected DNA molecule (here, also depicted as being coated with SSAP, although this may or may not be the case). Here, the torroidal trimer of Exo protein is shown to dissociate from the non-digested strand, although this may not necessarily be the case. The SSAP mediates displacement of the original complementary strand, and promotes annealing of the (SSAP-coated) complementary strand from the other DNA molecule until its 3'-terminus, or until the end of its sequence homology. Any 3'-ssDNA overhangs would not be digested by Exo, but by a host exonuclease with 3'-5' ssDNA exonuclease activity (not shown). The Exo protein 'trims' both the 5'-ssDNA overhangs via its ssDNA exonucleolytic activity, until only a nick remains, which is sealed by host DNA ligase. [file 1471-2199-12-16-S2.JPEG]

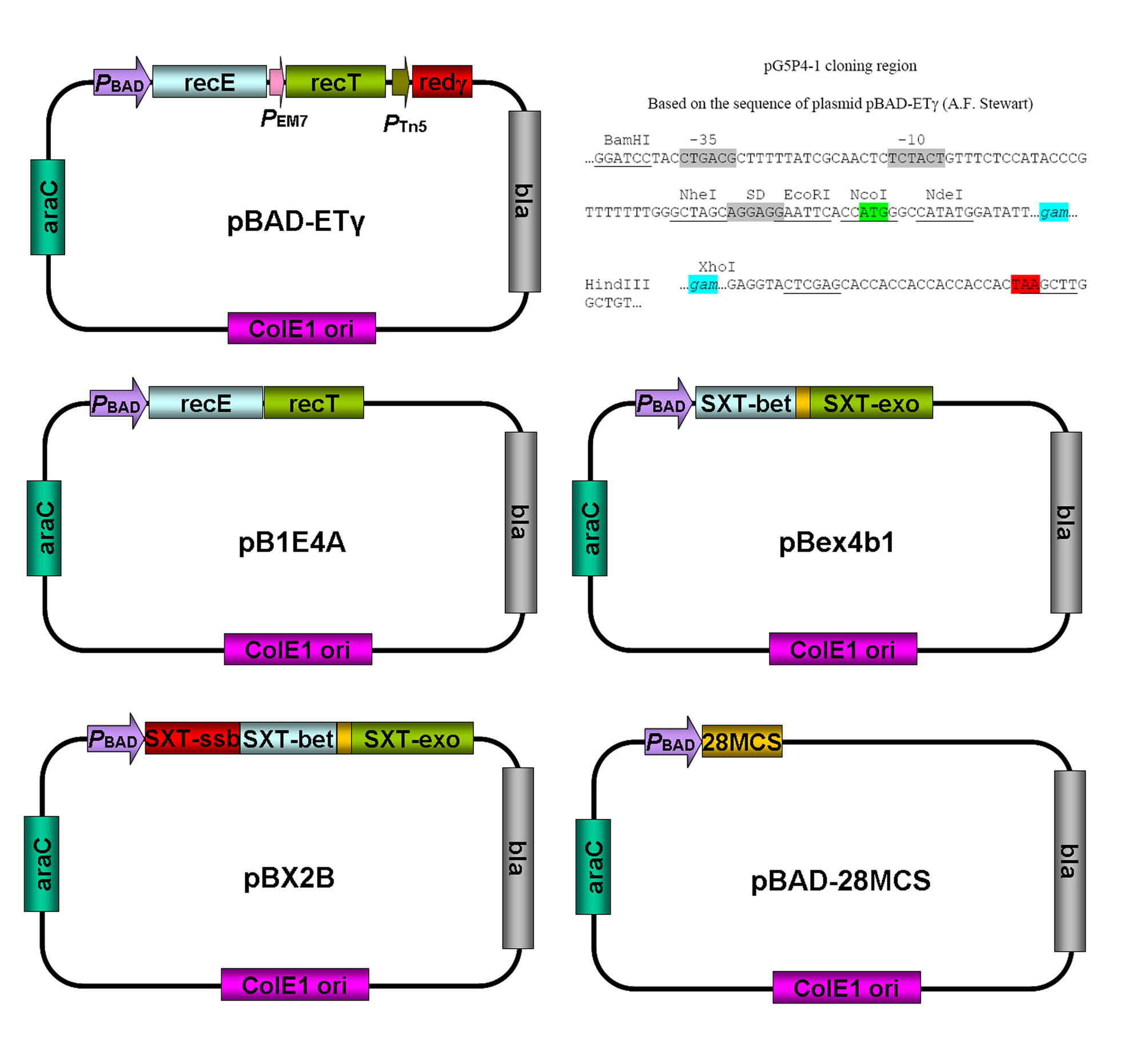

Supplement: Additional file 3 — Maps for the plasmids used in this study. [file 1471-2199-12-16-S3.JPEG]

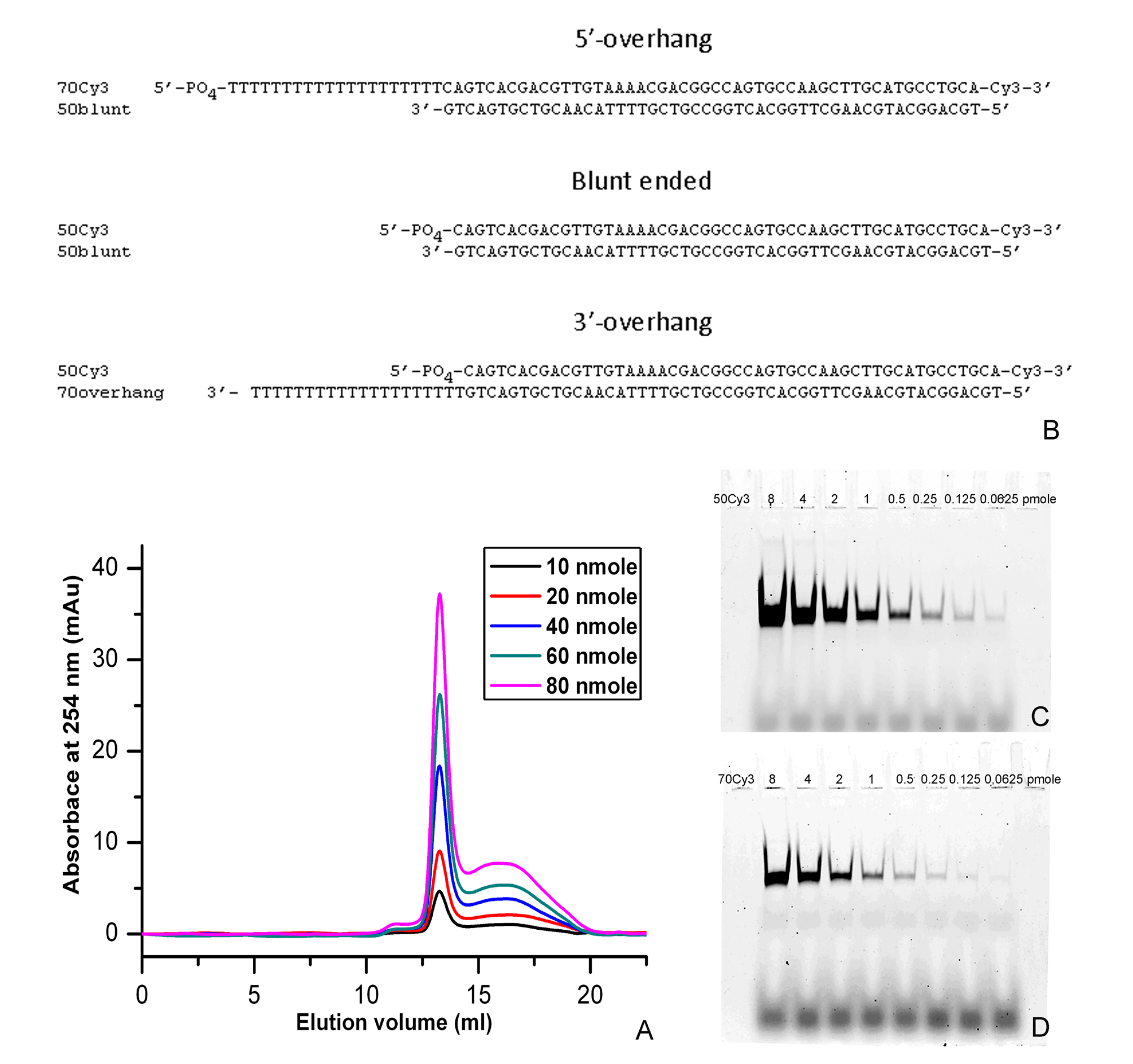

Supplement: Additional file 4 — Chromatograms of a range of concentrations of the dT75 oligonucleotide; fluorescence-scanned gel images of the 5'-phosphoryrated 50Cy3 and 70Cy3 oligonucleotides, and composition of the annealed oligonucleotide substrates used to characterize exonuclease activities. Panel A. Overlaid gel filtration chromatograms obtained for various concentrations (10-80 nmol) of the dT75 oligonucleotide used in the single strand exonuclease assays. Conditions used were identical to those described in the materials and methods section. Panel B: Composition of the 5'-overhang, Blunt ended and 3'-overhang substrates used to characterize the exonuclease activities of the SXT-Exo and lambda-Exo proteins. Panel C: fluorescence-scanned image of various concentrations of the 5'-phosphorylated-50Cy3 oligonucleotide (8 - 0.0625 pmol) resolved on a 7 M urea-TBE denaturing gel. Panel D: fluorescence-scanned image of various concentrations of the 5'-phosphorylated-70Cy3 oligonucleotide (8 - 0.0625 pmol) resolved on a 7 M urea-TBE denaturing gel. The band at the base of each lane corresponds to the dye used in the loading buffer. [file 1471-2199-12-16-S4.JPEG]
